# Clonal expansion of a virulent *Streptococcus suis* serotype 9 lineage distinguishable from carriage subpopulations

**DOI:** 10.1038/s41598-019-51576-0

**Published:** 2019-10-28

**Authors:** Niels Willemse, Kees C. H. van der Ark, Norbert Stockhofe-Zurwieden, Hilde Smith, Daisy I. Picavet, Conny van Solt-Smits, Henk J. Wisselink, Constance Schultsz, Astrid de Greeff

**Affiliations:** 1 Department of Global Health-Amsterdam Institute for Global Health and Development, Amsterdam UMC, Paasheuvelweg 25, 1105 BP Amsterdam, The Netherlands; 2 Department of Medical Microbiology, Amsterdam UMC, University of Amsterdam, Meibergdreef 9, 1105 AZ Amsterdam, The Netherlands; 3 Wageningen Bioveterinary Research, Wageningen University & Research, Houtribweg 39, 8221 RA Lelystad, The Netherlands; 4 EMCA Amsterdam, Department of Medical Biology, Amsterdam UMC, University of Amsterdam, Meibergdreef 9, 1105 AZ Amsterdam, The Netherlands

**Keywords:** Bacterial genes, Bacteriology, Infectious-disease diagnostics

## Abstract

*Streptococcus suis* is a porcine pathogen, causing severe invasive infections. *S. suis* serotype 9 is increasingly causing disease in Dutch and Chinese pig herds, but it is unknown whether all serotype 9 isolates are equally virulent and markers that can identify virulent strains are not available. Therefore, discrimination between virulent isolates and carriage isolates typically not associated with disease, is currently not possible. We collected tonsillar *S. suis* isolates from 6 herds not previously diagnosed with *S. suis* infections, and clinical *S. suis* isolates of previously diseased pigs. We confirmed the virulence of a virulent type strain and one representative clinical isolate, and the lack of virulence of two carriage isolates, in a pig infection model. Phylogenetic analysis of whole genome sequences of 124 isolates resulted in 10 groups, of which two were almost uniquely populated by clinical isolates. The population structure of *S. suis* serotype 9 appears highly diverse. However, analysis of the capsule loci sequences showed variation in a single region which fully correlated with a virulent genotype. Transmission electron microscopy suggested differences in capsule thickness between carriage and clinical genotypes. In conclusion, we found that that the *S. suis* serotype 9 population in the Netherlands is diverse. A distinct virulence-associated lineage was identified and could be discriminated based on the capsule locus sequence. Whilst the difference in virulence cannot be directly attributed to the DNA sequence, the correlation of capsule locus sequence with virulence could be used in the development of diagnostic tests to identify potential virulent *S. suis* serotype 9 in pigs.

## Introduction

*Streptococcus suis* is an important pathogen associated with a wide range of diseases in pigs including meningitis, arthritis and sepsis^1,2^, leading to major economic losses in the pig industry worldwide. Healthy pigs can carry *S. suis* asymptomatically on the mucosal surfaces of the tonsil, nasal cavities as well as the gastrointestinal tract^1,3,4^. Although serotype 2 is most often associated with clinical disease in pigs worldwide^5^ as well as with zoonotic disease in humans^6–8^, the proportion of serotype 9 among isolates from diseased pigs has increased substantially in the last two decades^9^. Nowadays, serotype 2 and 9 isolates are the most prevalent serotypes of invasive pig isolates in the Netherlands^10,11^. Zoonotic serotype 2 isolates generally belong to multi-locus sequence type (MLST) 1 and 7^10,12,13^, whereas zoonotic serotype 2 isolates belonging to ST20 diverged from serotype 9 isolates belonging to ST16^11^. Although serotype 9 isolates are considered non-zoonotic, a single case of serotype 9 infection in humans was reported in Thailand^14^.

Among serotype 2 isolates, virulent as well as less virulent isolates can be discriminated^15^. Virulent isolates of serotype 2 can be recognized by expression of muramidase-released protein (MRP) and extracellular factor (EF)^9,16^ and virulence is associated with a considerable number of putative virulence factors in *S. suis* serotype 2^17^. Moreover, several antigens were described as putative vaccine candidates^17,18^, although vaccines based on these antigens were not developed. The majority of the invasive serotype 9 isolates express a larger variant of MRP, but do not express EF^19^, whilst the virulence factor suilysin was shown to be expressed^20^. So far it is unknown whether virulent and less virulent isolates can be discriminated among serotype 9 isolates. In addition, antigens which generate a protective immune response against serotype 9 challenge, and which could be used for vaccine development, have not been described.

Given the large contribution of serotype 9 to *S. suis* related disease and outbreaks in pig husbandry, as well as its potential evolution into a zoonotic pathogen, a better understanding of the population structure of *S. suis* serotype 9 is urgently needed to improve diagnostics and control.

We studied *S. suis* serotype 9 isolates obtained from the tonsils of healthy pigs from farms without overt *S. suis* specific disease for over 1 year (carrier isolates) and isolates obtained from clinically diseased pigs (clinical isolates). We showed that a clinical serotype 9 isolate was capable to induce severe disease in pigs after experimental infection, thus reconfirming its virulence, whereas carrier isolates did not cause severe disease. This indicates the presence of different pathotypes among *S. suis* serotype 9 isolates, which we further investigated using whole genome sequencing. We observed a diverse population structure among carriage serotype 9 isolates, which was previously unknown, whilst clinical isolates belonged to a single clonal expansion. Finally, we describe differences in the capsule loci between carriage and clinical isolates and propose the *cpsK* gene as a diagnostic target for the detection of virulent serotype 9 isolates.

## Results

### Sample collection

Thirty-two clinical isolates, cultured from the brains of diseased pigs from 32 different unrelated pig herds in the Netherlands, as part of standard veterinary diagnostic procedures at the laboratory of the GD Deventer, were included. An additional 16 clinical serotype 9 isolates from the collections of consortium partners were included into the study, as well as 28 clinical serotype 9 isolates from our previous study^11^ and isolate 8067, which is a well-studied virulent isolate^9^. Carrier isolates were obtained from 6 unrelated farms, with multiple isolates per farm and a maximum of one isolate per animal (Supplementary Table 1). To collect carriage isolates, tonsil swabs of 50 sows and 223 piglets from 6 different farms in the Netherlands which did not experience *S. suis* disease during the previous 12 months, were analyzed by using a *S. suis* serotype 9 specific PCR. Pig breeds included different crossbreeds of Dutch Landrace with Yorkshire (Farms B_2, B_4, B_5, B_7), Finnish Landrace with Yorkshire (Farm B_3) and English Landrace with Large White (Farm B_6) which are all commercial breeds commonly used in the Netherlands. The number of sows in these farms varied between 196 and 800, which are common pig herd sizes in the Netherlands. A total of 36 out of the 50 sows (72%) and 106 out 223 (48%) piglets tested were positive in a serotype 9 specific PCR, with varying fractions of positive tonsil swabs between the farms (Supplementary Table 2). These data clearly indicate that high percentages of the piglets and sows carried *S. suis* serotype 9 isolates on their tonsils on farms without overt *S. suis* disease during the 12 months prior to sampling. Forty-six carriage isolates were selected for this study. One additional carriage isolate was provided by a consortium member from France. Taken together, 47 carriage isolates and 77 invasive isolates were used in this study (Supplementary Table 1).

### Virulence of serotype 9 isolates

Isolates were defined as virulent if grown from sterile sites (blood, cerebrospinal fluid, brain) or in pure culture from pigs with clinical disease suspect of *S. suis* infection. Thus, clinical isolates included in this study are considered virulent. The virulence of serotype 9 isolates was confirmed in an infection experiment in piglets using two randomly selected carrier isolates obtained from the tonsils of healthy pigs of two different herds (21853 and 21900) and one clinical isolate (21970). Serotype 9 strain 8067, a clinical isolate previously used in experimental infections in piglets^20^ was used as a positive control. Caesarean derived, colostrum deprived (CDCD) piglets at 6 weeks of age were inoculated intravenously with a dose of 5·10^7^ CFU and monitored for 8 days.

**Figure 1:**
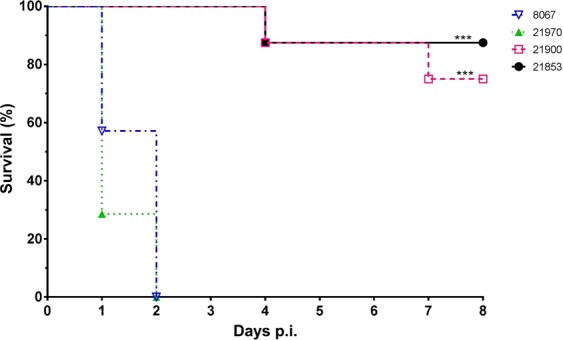
Kaplan-Meier survival curve of experimental infection of 30 pigs with two carriage isolates (21853 and 21900) and two clinical isolates (21970 and 8067). Pigs were randomly allocated to one of four groups and intravenously inoculated with 1 ml of PBS containing 5∙10^7^ CFU. Groups infected with clinical isolates consisted of 7 pigs, whilst groups infected with carriage isolates consisted of 8 pigs. All pigs in both groups inoculated with the clinical isolates died or were euthanized after two days. In contrast, only 1 or 2 pigs inoculated with the carriage isolates died before the end of the experiment, but after at least 4 days after inoculation. ***P < 0.001 (Mantel-Cox Log-rank test).

**Figure 2:**
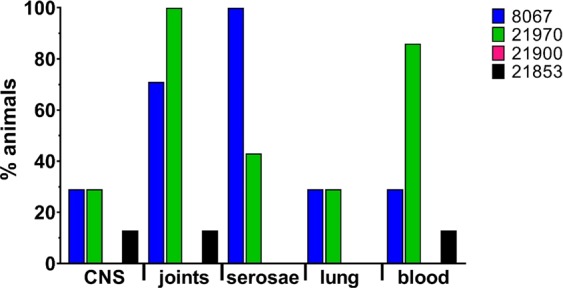
Percentage of animals from which *S. suis* could be isolated from the indicated organs postmortem. *S. suis* was isolated from CNS (meninges), one or several joints, serosae (peritoneal, pleural or pericardial), lungs and blood at a higher percentage of pigs inoculated with the clinical isolates 8067 (n = 7) and 21970 (n = 7) than from piglets inoculated with the carriage isolates 21900 (n = 8) and 21853 (n = 8). Each bar represents a group of pigs inoculated with one of the experimental *S. suis* isolates.

**Figure 3:**
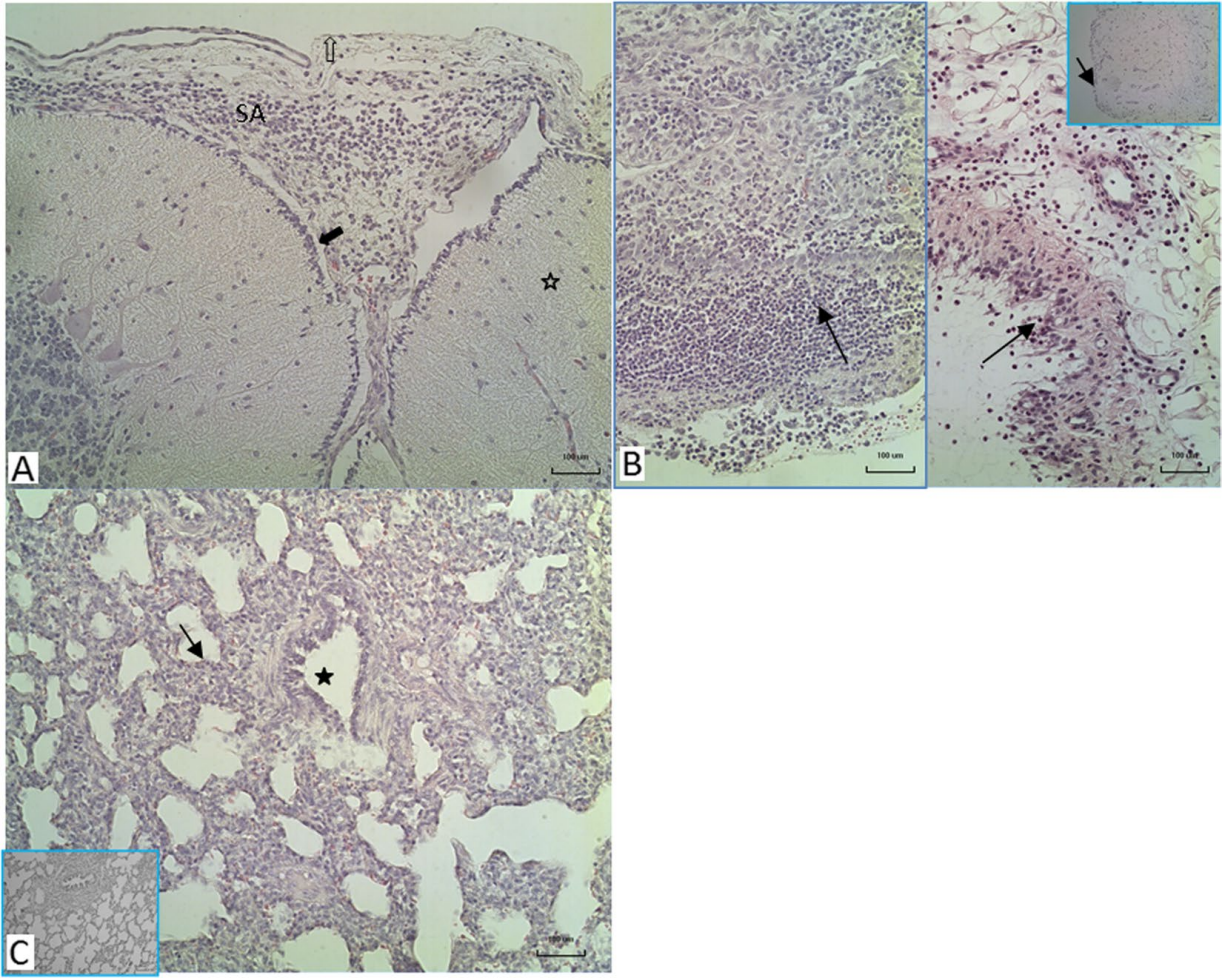
Histopathological findings in the brains, joints and lungs of pigs infected with clinical isolates of S. suis type 9. (**A**) Meningitis of the brain, here cerebellum (asterisk) consisting of infiltration of leucocytes and fibrin in the subarachnoidal space (SA), pia (solid arrow), arachnoid membrane (open arrow). (**B**) Various degrees of inflammation of the synovial membrane after infection ranging from severe fibrinous-suppurative inflammation (left part) to a mild to moderate mixed leucocyte infiltration in the intima (arrows) and subintima of the synovium, (inset: normal appearance of the synovium). (**C**) Acute interstitial pneumonia with lymphocyte and macrophage infiltration in the alveolar septae (arrow), no changes in the bronchioles (asterisk), (inset: normal histological appearance of lung); H&E staining ×10 objective magnification.

All 14 animals inoculated with the clinical isolates were euthanized within two days after inoculation because of serious signs of a *S. suis* infection, mainly arthritis and meningitis (Fig. 1). In contrast, 3 out of 16 piglets inoculated with the carrier strains were killed 4 and 7 days after inoculation with clinical signs of arthritis. The mean number of days until death of diseased pigs was 1.6 and 1.3 for animals inoculated with the clinical strains 8067 and 21970 and 7.6 and 7.5 days for the carrier strains 21853 and 21900, respectively (Table 1). Significant differences were observed in the number of specific clinical signs of disease between piglets inoculated with the clinical isolates and with the carrier isolates obtained from healthy piglets (Supplementary Fig. 1). *S. suis* was isolated at significantly higher frequency from the joints and meninges of piglets inoculated with the clinical isolates than after inoculation with isolates from healthy pigs (Fig. 2). Gross pathology findings revealed an acute arthritis due to inflammation of the synovial membrane or peri-articular soft tissues in one or more joints in all pigs (100%) of both groups inoculated with clinical isolates and 3 pigs (18%) of the groups inoculated with the carrier isolates. The histological findings in the synovia were consistent with *S. suis* typical inflammation ranging from diffuse mild inflammation to severe fibrinopurulent inflammation of the synovial membrane (Fig. 3). No inflammation of the serosae of the body cavities was seen in the pigs infected with carrier isolates, but this was found in 10 of 14 pigs (70%) infected with clinical strains. Typical histological changes in the brains consisted of inflammation of cerebral and cerebellum meninges varying from diffuse interstitial edema and congestion to fibrinopurulent meningitis and incidentally meningoencephalitis, which was observed in 29% of all pigs of both groups infected with clinical isolates, but in none of the pigs infected with the carrier isolates. In addition, only in the groups inoculated with clinical isolates an acute interstitial pneumonia with diffuse hyper cellularity of alveolar septae was observed in more than 70% of the pigs (Supplementary Fig. 1B).

**Table 1:** Summary of the results from the virulence study as presented in Figs 1 and 2.

*S. suis* isolate	No. of pigs	Mortality (%)	Mean No. of days until death	Specific symptoms No. obs/total No. obs
21853	8	13	7.6	4/128
21900	8	25	7.5	11/128
21970	7	100	1.3	20/22
8067	7	100	1.6	25/36

### Bacterial population structure

Forty-seven *S. suis* serotype 9 isolates collected from farms without serotype 9 infections were sequenced together with 77*S. suis* serotype 9 isolates obtained from diseased pigs to determine the population structure and to investigate the genetic differences between carriage and clinical isolates. Draft assemblies of the isolates were annotated using the Prokka^21^. The clinical isolates demonstrated significant larger genome sizes compared to the carriage isolates (Supplementary Fig. 2). The annotation files were further used as input for Roary^22^, which created homology groups based on 95% identity at the amino acid level. The resulting core genome consisted of 1149 genes and the pangenome consisted of 6787 genes. A core genome alignment was used as input for a Bayesian Analysis of Population Structure (BAPS) to cluster the isolates into population groups. BAPS identified 10 clusters with a set maximum of 20 clusters (Supplementary Fig. 3). Out of 77 clinical isolates, 73 (95%) isolates clustered into BAPS group 2. Of the remaining 4 isolates, 3 isolates clustered into BAPS group 4 and 1 isolate clustered into BAPS group 8. BAPS group 8 is a residual group consisting of 3 isolates that are not similar to each other, but could also not be clustered in any other BAPS group. We therefore designated BAPS groups 2 and 4 as the invasive population groups. The 47 carriage isolates were more diverse as they clustered into 9 BAPS groups, and only BAPS group 4 did not contain carriage isolates (Supplementary Fig. 4). MLST Sequence types (STs) were extracted from the sequencing reads, which resulted in 14 novel STs of which 10 STs belonged to carriage isolates (Supplementary Table 3). The diversity of STs among the carriage isolates was consistent with the diversity of BAPS groups.

**Figure 4:**
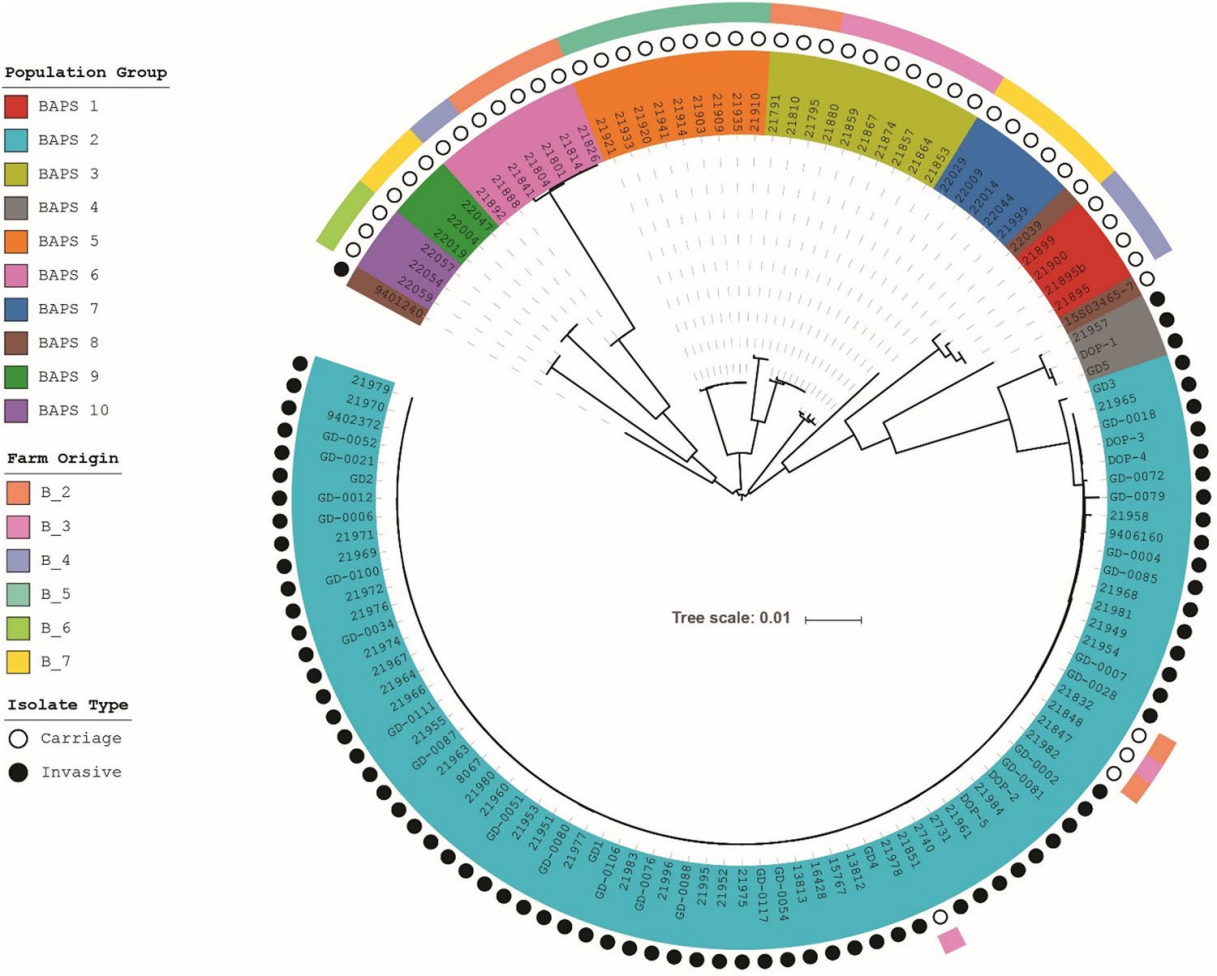
Phylogenetic relationship between invasive and carriage isolates. The BAPS group colors are highlighted on the isolate’s name. The second ring of dots indicates the phenotype of the isolates as invasive (black) or carriage (white). The colors on the outer ring represent the farms from which the carriage isolates have been collected.

A phylogenetic tree of the core genome was created using RAxML^23^ to understand the relationships between the population groups (Supplementary Fig. 5). The invasive BAPS population groups 2 and 4 clustered near each other in the tree, whilst other population groups branched away from the invasive isolates revealing the population structure of serotype 9 isolates included in this study. BAPS group 8 consisted of 3 isolates, which did not group together in the tree indicating that these isolates are outliers in the serotype 9 population structure. The population tree was overlaid with the origin of the farms from which the carriage isolates were collected (Fig. 4). A correlation between BAPS groups and the farm origin was observed. Each carriage BAPS group was found to be associated with one or two farms only. Additionally, farms B_2 and B_3, without disease in the past 12 months, each had two isolates present belonging to invasive BAPS group 2. The virulent isolates used in the infection experiment were confirmed as representatives of the main clonal cluster of BAPS group 2 in a phylogenetic analysis of this group (Supplementary Fig. 6)

### Capsule locus analysis

**Figure 5:**
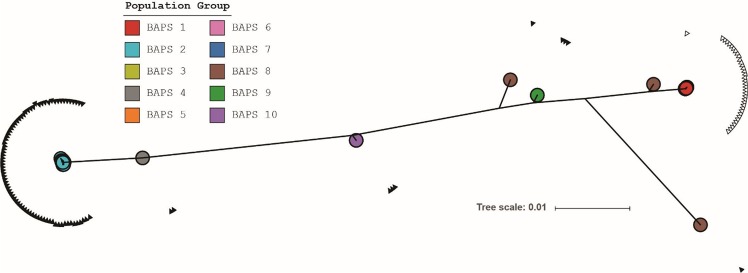
Phylogenetic tree based on the concatenated nucleotide alignment of capsule genes *cpsA-cpsN*. For clarity, names of the isolates are omitted. Colored circles indicate the BAPS population groups to which an isolate belongs. BAPS groups 1, 3 and 5–7 are highly similar and are located under the red circle of BAPS group 1. A filled or empty triangle respectively represents the presence or absence of a deletion between the *cpsN* and *glf* genes.

To investigate the relation between the single clonal expansion of invasive isolates and genomic virulence determinants we extracted the regions corresponding to the capsule genes from the various BAPS groups. The capsule locus ranged from *cpsA* (*wzg*; SSUD12_1370 in *S. suis* D12) until the *glf* gene (UDP-galactopyranose mutase; SSUD12_1356 in *S. suis* D12^24^). The genes were concatenated to form a nucleotide alignment, which was used to create a phylogenetic tree (Fig. 5). The structure of the tree differs from the structure of the tree generated from the core genome alignment (Supplementary Fig. 5). BAPS groups 1, 3, 5, 6 and 7 containing carriage isolates clustered together and opposite to the virulent BAPS groups 2 and 4. BAPS groups 8, 9 and 10 containing carriage isolates as well, are intermediates. Again, isolates of BAPS group 8 did not cluster together, and are outliers in their capsule locus as well as in their core genome.

**Figure 6:**
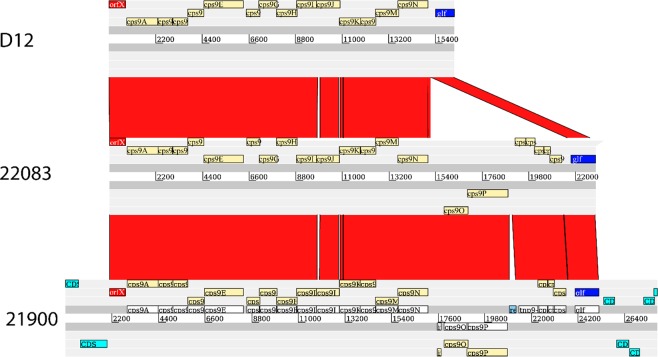
Comparison of the serotype 9 capsule loci of isolates D12, 22083 and 21900. Both D12 and 22083 were annotated by Prokka, whilst 21900 was annotated previously^24^. The capsule loci are similar except for the deletion after *cpsN*. The deleted area contains cps9O and cps9P, which may form a methyltransferase.

However, an analysis of the capsule locus did reveal that only two different capsule loci were present across BAPS population groups (indicated by filled and empty triangles in the phylogenetic tree in Fig. 5). More detailed investigation of these two capsule loci showed high similarity in gene content except for a deletion in the capsule locus of the invasive isolates before the *glf* gene and after *cpsN* (*wzx* or a putative flippase; SSUD12_1357 in *S. suis* D12) (Fig. 6). This deleted region encodes an integrase fragment, transposases, hypothetical proteins, and a Type I specificity (S) subunit and a Type I modification (M) subunit. The deletion is significantly associated with the invasive BAPS groups 2 and 4 (80/80) compared to the non-invasive BAPS groups 1, 3, 5, 6, 7, 9 and 10 (8/41); p-value < 0.001 (Pearson’s Chi-squared test).

The proteins translated from the capsule locus of the various isolates were also individually compared to determine if certain genes differed more than others from the average difference. The genes for comparisons were chosen from isolates GD2, as a representative of virulent ST16 clonal complex isolates, and 21853, as representative of carriage isolates. The genes were compared at the protein level and the amino acid (aa) identities were plotted for each protein (Supplementary Fig. 7). The CpsA and CpsK proteins had less than 95% identity, and are therefore most discriminative between the two loci. Blast identified CpsA as a LytR-CpsA-PSR (LCP) protein containing two domains: DNA_PPF or DNA polymerase processivity factor (pfam02916) and LCP (pfam03816). The domain with the lowest amino acid identities between the two proteins was the DNA_PFF domain. CpsK was identified as a capsular polysaccharide synthesis protein with domain pfam05704. The largest differences in amino acids could be found between aa 200 and 250 (Supplementary Fig. 8). This deviant region is present in all isolates in BAPS groups 2 and 4.

A BLAST search was performed to investigate the association of the deviant region in the CpsK protein with clinical isolates. The search was done by aligning the invasive isolate GD2 CpsK complete protein with the NCBI database, which identified a few proteins with over 80% identity (Table 2). The majority of sequences with a high identity originate from invasive serotype 9 isolates contributed from our previous study^11^ and were included in this study and therefore excluded from Table 2. The foreign isolates with 99% amino acid identity had invasive as well as carriage phenotypes attributed to them and included isolates from China and the UK, as well as 3 isolates (LSS92, LSS70 and SS981) with unknown phenotypes.

**Table 2:** List of foreign serotype 9 isolates not included in this study with their amino acid identities with CpsK of isolate GD2.

*S. suis* isolate	Identity (%)	Phenotype	Country	Reference
LS2N	99	non-clinical	United Kingdom	Weinert *et al*.^26^
LSS92	99	NA	NA	NA
D12	99	pneumonia	China	Zhang *et al*.^26^
LS0P	99	non-clinical	United Kingdom	Weinert *et al*.^26^
S91K	99	systemic (brain)	United Kingdom	Weinert *et al*.^26^
DN13	99	NA	China	NA
GZ0565	99	meningitis	China	Wu *et al*. (2007)
LSS70	99	NA	NA	NA
SS981	99	NA	NA	NA
22083	94	NA	NA	Okura *et al*.^24^
LS5Y	94	non-clinical	United Kingdom	Weinert *et al*.^26^
LS6K	94	NA	United Kingdom	Weinert *et al*.^26^
LSS55	94	NA	NA	NA
LSS66	94	NA	NA	NA

For 6 matches, the amino acid identity was 94%. These matches included strain 21853 and serotype 9 reference strain 22083^25^. For these 6 isolates, the phenotype was unknown for 4 isolates and non-clinical for one isolate. Finally, 9401240 is a continuous outlier with its invasive phenotype in this study as well as the previous^11^. The known phenotypes of previously submitted serotype 9 strains are limited, but when present these metadata corroborate our phenotypical observations on virulence and carriage.

The differences observed in the capsule locus between the invasive and carriage isolates prompted us to look for differences in the capsule itself by means of Transmission Electron Microscopy (TEM). Carriage isolates 21900 and 21853 and invasive isolates 8067 and GD2 were selected for TEM (Supplementary Fig. 9). These isolates were also used in the virulence *in vivo* experiment except for isolate GD2, which was chosen instead of 21970 to include an ST16 isolate. The cps thickness of carriage isolates when fixated in McDowell’s fixative was 20.5 ± 7.1 nm and the thickness of invasive isolates 20.9 ± 6.4 nm. This difference was not significant (p = 0.81). However, when lysine-acetate was added to the fixative, it was impossible to measure the cps thickness of carriage isolates 21853 and 21900 due to absence of the layer, while the thickness of virulent isolates was 32.9 ± 15.4. No differences were observed between the two fixatives for the positive control *S. suis* S10 and negative control non-encapsulated *S. suis* J28.

## Discussion

We performed a systematic analysis of carriage isolates from the tonsils of healthy pigs and clinical *S. suis* serotype 9 isolates in the Netherlands. The clinical isolates obtained from individual diseased pigs clustered mainly together in one phylogenetic group (BAPS group 2), whereas the carriage isolates, which originated from 6 different herds, were distributed across 8 different groups. The clinical isolates had higher gene counts, which is in contrast to previous findings, where invasive isolates of multiple serotypes had smaller genomes than carriage isolates^26^. These data could reflect the difference in virulence between the isolates belonging to the two different clusters, which was confirmed with representative isolates in an infection experiment. The results of this experiment indicated that the virulence of the tested clinical isolates and tonsillar isolates from healthy pigs differed significantly.

Although *S. suis* serotype 9 isolates are nowadays very frequently isolated from diseased pigs in the field, serotype 9 isolates were under experimental conditions shown to be less pathogenic compared to serotype 2 isolates^20,27^. Still, intravenous infection with high dose of *S. suis* serotype 9 (ST16) induced mortality and specific clinical symptoms^28^. In this study, we used highly susceptible CDCD piglets to compare the pathogenicity of the clinical and tonsillar serotype 9 isolates. By using an infection dose of 5·10^7^ CFU, clear signs of *S. suis* disease were induced in the pigs after inoculation with virulent isolates, whereas only minor signs of *S. suis* disease were induced by the carrier isolates. The clinical course after infection with the virulent isolates, but not with carrier isolates, and the pathological findings in the meninges, the synovial membranes and the serosae, are in line with those observed under field conditions and experimental conditions. Whether *S. suis* is an important cause of pneumonia in pigs remains under debate and so far, clear experimental evidence in support is still lacking. Interestingly, in this study, in 70% of diseased pigs an acute, interstitial pneumonia with increased numbers of macrophages and lymphocytes in the alveolar wall, but no signs of an exudative broncho-pneumonia, was observed. These findings may be related to the occurring bacteremia, which may facilitate subsequent localized infections or secondary infection with other bacterial pathogens.

Here we analyzed a large collection of serotype 9 isolates from the Netherlands and revealed that these isolates belonged to a total of 21 different STs. Ten STs were associated with the clinical phenotype and 11 STs with the carriage phenotype. All together 14 new STs were determined amongst carriage isolates. Of all clinical isolates, 95% belonged to ST16 or a SLV of ST16. These results clearly indicate that isolates belonging to ST16 are mainly responsible for the disease caused by serotype 9 infections among pigs in the Netherlands. Thus far, clinical invasive serotype 9 isolates collected in the Netherlands were associated with ST16^10,11^, whereas in Asia serotype 9 isolates were very diverse and associated with a range of singleton STs^29^. Our data demonstrates that clinical isolates from The Netherlands have a different population structure than serotype 9 isolates from China. Interestingly, 2 out of 7 farms (B_2 and B_6) which were sampled to collect carriage isolates, had disease outbreaks caused by serotype 9 isolates after finishing the collection of these samples (personal communication from farmers). Interestingly, we showed that piglets sampled at farms B_2 and B_3 did carry ST16 isolates of the virulence associated BAPS group 2 (isolates 21832, 21848, 21847, and 21851) at the time of sampling. Unfortunately, we do not know whether the reported infections in farm B_2 were caused by (a SLV of) a ST16 isolate. Therefore, we cannot conclude whether these four isolates are asymptomatically carried virulent isolates or non-virulent isolates. The only non-novel ST among the carriage isolates, ST48, was found on farm B_6. According to the MLST database, ST48 was isolated in 1998 from the brain of a pig with meningitis in the UK (Y02316). This suggests that under certain conditions, ST48 isolates can be virulent. Unfortunately, it is not known which ST caused problems on farm B_6 after tonsil sampling.

Recently, Dong and co-workers compared 30*S. suis* serotype 9 isolates mainly obtained from China and Vietnam by MLST^29^. The isolates were designated to two different clusters based on the presence/absence of 23 different virulence-related genes. However, using a mice model, no difference in virulence was observed between isolates belonging to the two different clusters, further demonstrating the potential weakness of mouse models to assess virulence of a porcine pathogen.

Whole genome sequencing of invasive and carriage isolates from this study revealed that the average genome size of the virulent isolates was larger compared to the genome size of carriage isolates. This finding is in contrast to previous findings, where more virulent isolates had smaller genomes^26^. The genomes of the serotype 9 isolates contained two different capsular loci, which were correlated with virulent or carriage isolates respectively (Figs 5 and 6). Interestingly, the carriage isolates showed much more similarity with each other when comparing them on the capsule locus, than compared to the core genome, which suggests a higher conservation of the capsular locus compared to the core genome. The two different capsular loci were previously described for serotype 9 isolates 22083 and D12^24^. Isolate 22083 is the serotype 9 reference isolate with an unknown clinical phenotype, whereas D12 was an invasive isolate causing pneumonia in a pig from China. Compared to isolate 22083, the capsular locus of isolate D12 contains a deletion^24^. We here show that a carriage-associated capsule locus was identified in isolate 22083, whereas an virulence-associated capsule locus was identified in *S. suis* D12. Compared to most of the genes in the capsule locus, the fragment absent in the virulence-associated capsule locus contained genes transcribed in the reverse direction, encoding a Type I S and Type I M subunit. This indicates that a methyltransferase could be missing in the virulent genomes. Methylation patterns of the genome can be important for overall transcription levels^30^, but regulatory capabilities due to rapid switching as typical for type I restriction-modification systems are unlikely to explain the phenotype due to the lack of nearby additional Methylase_S domains^31,32^.

The most dissimilar genes in the capsule locus are *cpsA* and *cpsK* (Supplementary Fig. 7). CpsK is a capsular synthesis protein. The structure of the serotype 9 capsular polysaccharide was recently determined and the proposed function of CpsK is to transfer the last rhamnose to the glucitol^33^. We postulate that the *cpsK* gene is an interesting diagnostic target as the region between 600 and 750 nucleotides is highly different between the virulent BAPS 2 isolates and the carriage BAPS groups. The *cpsK* gene is also highly specific for serotype 9*S. suis* isolates as was shown by blast search. Protein blast hits with an identity over 80% included isolates from the UK that were previously classified as non-typable^26^. However, their genome sequences contained a serotype 9 capsule locus, which was consistent with the recently released *S. suis*_serotyping pipeline^34^. The function of CpsK in S*. suis* serotypes 1, 1/2, 2, and 14, was recently elucidated, and found to be crucial in the determination of the serotype. A single amino acid substitution resulted in a serotype switch from serotype 1 to 14 or 2 to 1/2^35^.

Both the thickness and composition of the polysaccharide capsule are associated with virulence^36^. A visualization of the capsule layer by TEM did not reveal any differences in capsule thickness when McDowell’s fixative was used. However, after addition of lysine acetate, a clear difference was observed in the thickness of the capsule layers. The observed differences upon lysine acetate addition to the fixative could indicate a difference in capsule composition. The diamine lysine acetate aids in the fixation of hydrated structures, such as cps^37^. The influence of lysine acetate on the fixation of *S. suis* cps differs per serotype^38^. These results indicate a difference in capsule layer, but to what extend and how difference in the thickness of the capsule layer mediates virulence remains to be investigated.

With this study, we significantly broadened the knowledge on the important, but highly understudied *S. suis* serotype 9. We demonstrated a previously unseen depth in the population structure among carriage isolates of *S. suis* serotype 9 in The Netherlands. Two pathotypes were discriminated in an experimental infection model and studied by whole genome sequencing. Our analyses identified genomic signatures in the capsule locus that could differentiate between virulent and carriage *S. suis* isolates. This would potentially allow the development of a discriminatory diagnostic test which can aid in reducing the burden of *S. suis* serotype 9 related infections on pig farms. Future vaccine development strategies maybe therefore be focused on the virulent isolates exclusively. Such improved diagnostics and preventive vaccination can help in combatting *S. suis* infections in the field.

## Methods

### Sample collection

(1)*S. suis* **serotype 9 strains from clinically diseased piglets**. The GD Animal Health (Deventer, The Netherlands) kindly provided *S. suis* serotype 9 isolates obtained from the brains of diseased piglets. The 32 clinical *S. suis* isolates used in this study were obtained from 32 different pig herds in the Netherlands. An additional 16 clinical serotype 9 isolates from the collections of the partners were included. Moreover, 28 clinical serotype 9 isolates from our previous study^11^, as well as virulent isolate 8067^9^ were included into the study (Supplementary Table 1).

(2)*S. suis* ***serotype 9 strains from tonsil samples of healthy pigs***. Seven pig herds in the Netherlands, which did not use *S. suis* specific autovaccines nor used feed medication and where pigs did not show signs of meningitis, arthritis, or sudden death for over 1 year were selected. Tonsil swabs from sows and piglets (9–10 weeks of age) were collected as described before^39^. Swabs were transferred to the laboratory at 4 °C in 2 ml Todd–Hewitt broth with 0.25% Streptococcus Selective Supplement (Oxoid) and 0.2 μg/ml cristalviolet. To elute the bacteria swabs were subsequently sonicated in a water bath (Ultrasonic Cleaner, VWR symphony) at room temperature for 90 min. The material was then stored at −70 °C in the presence of 15% glycerol.

A serotype 9 specific PCR was used to identify tonsil samples positive for *S. suis* serotype 9 isolates^40,41^. Therefore 200 µl of the tonsil material was incubated overnight at 37 °C in 1.5 ml Todd Hewitt Broth containing 0.25% Streptococcus Selective Supplement (Oxoid) and 0.2 μg/ml cristalviolet. Fifty µl of the samples was subsequently used for DNA isolation^41^.

Tonsil samples positive in the PCR were used for isolation of *S. suis* serotype 9 isolates. Therefore, tonsil samples were plated on Columbia blood agar plates supplemented with 6% horse blood, 0.25% Streptococcus Selective Supplement (Oxoid) and 0.2 μg/ml cristalviolet. Plates were incubated overnight at 37 °C. Colonies were lifted onto sterile GeneScreen Plus membranes (New-England Nuclear Corp., Boston, USA) and hybridized to a 32P-labeled serotype 9 specific probe. Hybridizing colonies were picked from the original plates and used for further characterization and typing.

### Experimental infection in pigs

Thirty caesarean-derived and colostrum deprived (CDCD) piglets (breed: Topigs 20) were obtained from 6 sows of a herd with a high health status and free of relevant pig pathogens. Piglets were produced and raised by WBVR housed at the animal facilities of WBVR with ad libitum access to water and feed. Three days before the inoculation the pigs were transferred to the final animal rooms. Animals were randomized by sex and weight and allocated to two groups of 7 and two groups of 8 piglets. At the age of 6 weeks the animals were injected intravenously with 1 ml of PBS containing 5 * 10^7^ colony forming units (CFU) of *S. suis* serotype 9 isolates. To monitor the health status of piglets, body temperatures and clinical scores were systematically recorded twice a day, starting at 1 day before inoculation to record baseline levels. Piglets were followed clinically twice daily with special regard to signs of meningitis and arthritis. Blood was collected from the jugular vein at the day before inoculation and days 4, 6 and 8 after inoculation to monitor white blood cell counts (WBC) and bacteremia. WBCs were counted using an automated cell counter (Sysmex, pocH-100iV-diff).

### Pathology

Pigs were sacrificed at 8 days post-infection or when pigs reached predefined humane end points and gross pathology performed with special emphasis on changes in joints of all limbs, meninges of cerebrum and cerebellum, serous surfaces of abdomen and thorax cavity, heart lung, spleen, liver and kidneys. From all of these organs/sites samples were taken by disposable inoculation loops for bacteriological examination. Tissue specimens from several CNS sites, heart, lung, joint capsules, liver, kidneys and spleen were formalin fixed for histological examination. Formalin fixed organ material was embedded in paraffin, sectioned at 3–5 µm, and subsequently stained with haematoxylin and eosin. Sections were microscopically screened for pathological changes.

### Whole genome sequencing

The selection of isolates for sequencing consisted of 46 carriage isolates and 32 invasive isolates from the sample collection phase of this study. Companies in our consortium contributed an additional 16 invasive serotype 9 isolates and one carriage isolate from France. Finally, the 28 invasive serotype 9 isolates from our previous study^11^ were also included, as well as invasive isolate 8067^9^ (Supplementary Table 1).

Bacterial isolates were sequenced by paired-end HiSeq sequencing with 2 * 125 basepairs. Adapters were trimmed from the reads using cutadapt (https://cutadapt.readthedocs.io/en/stable/) and the reads were further trimmed for quality by sickle (https://github.com/najoshi/sickle) with a cutoff Phred score of 20. Assemblies were constructed using SPAdes 3.9.0^42^ using the long Illumina paired reads (2 * 150 basepairs) settings including the–careful setting. Contigs with <10 coverage and/or <500 basepairs were excluded from further analyses. Assembly statistics have been added in (Supplementary Table 4) and sequencing reads and assemblies have been submitted to the European Nucleotide Archive.

### Genomic analysis

Serotype and sequence type of the isolates were obtained using SRST2 0.2.0^43^ and the Ssuis_serotypingPipeline^34^. Isolates not belonging to the *S. suis* species either due to lack of a RecN gene or isolates that expressed serotypes other than serotype 9 were excluded from further analyses. Novel alleles and novel sequence types were submitted to the *S. suis* MLST database (http://pubmlst.org/ssuis/). Prokka 1.9 (https://github.com/tseemann/prokka) was run on the draft assemblies and the GFF output files were fed into Roary 3.6.8^22^ with default settings. Roary clustered the genes into homology groups creating a core genome and a pangenome. The core genome alignment from Roary was used by BAPS 6.0^44^ (in particular hierBAPS) to cluster the isolates into population groups with a single level of hierarchy and a prior upper boundary of 20 clusters (Supplementary Fig. 3). All phylogenetic trees were created using RAxML 8.1.6^23^ with the GTRGAMMA substitution matrix and with as many bootstraps until the bootstopping criterion was reached. Best trees were visualized using iTOL v3^45^. Annotated genomes were viewed using Artemis 16.0.0^46^ and genomic comparisons (e.g. capsule loci) were performed using ACT 13.0.0^47^.

### Transmission electron microscopy

Bacteria were grown overnight in Todd Hewitt broth with 5% Yeast extract. The next morning, a growth curve was started and the bacteria were collected in log phase (OD = ~0.7). Bacteria were immediately prefixed in 1% glutaraldehyde, 4% paraformaldehyde in 0.1 M sodium cacodylate (Merck) with or without 1.5% (w/v) lysine acetate. After fixation, the samples were washed in 0.1 M phosphate buffer followed by a washing step in distilled water, osmicated for 60 minutes in 1% OsO4 in water and washed again in distilled water. Washed bacteria were subsequently dehydrated through a series of ethanol’s and embedded in resin LX-112 (Ladd). The resin blocks were polymerized for 48 hours at 60 °C. Ultrathin sections of 80 nm were cut on a Reichert EM UC6 with a diamond knife, collected on formvar coated grids and stained with uranyl acetate and lead citrate. Photographs were taken on a Tecnai electron microscope with a Veleta camera. Image analysis and measurements were performed with ImageJ (Version 1.52e). Images were obtained from 2 independent replicates of each isolate. A random selection of 8 bacteria of each replicate was measured, resulting in 16 measurements per isolate and 32 for each group of carriage and invasive isolates. A t-test was performed to determine significance of the differences. For bacteria fixed in McDowell’s with lysine acetate, 10 bacteria of each isolate were analyzed for the presence of a capsule layer.

### Ethics statement

The established principles of laboratory animal use and the EU and Dutch laws related to animal experiments were adhered to in this study. The Dutch Commission for Animal Studies approved the project”Vaccine development to combat *Streptococcus suis* infections” under number AVD401002015140. The animal experiment was approved by the Ethical Committee of Wageningen Bioveterinary Research (The Netherlands), in accordance with the Dutch law on animal experiments (permit number 2016054).

## Data Access

Sequencing data for this study have been deposited in the European Nucleotide Archive (ENA) in study accession: http://www.ebi.ac.uk/ena/data/view/PRJEB20548. Assemblies for isolates can be found under accession numbers as indicated in Supplementary Table 4. Additional metadata of the sequenced isolates can be found in Supplementary Table 4.

## Supplementary information

Supplementary Figures and Tables
