# Supplementary material for: Clonal expansion of a virulent Streptococcus suis serotype 9 lineage distinguishable from carriage subpopulations
Source: Sci Rep. 2019 Oct 28;9:15429. doi: 10.1038/s41598-019-51576-0 (PMC6817849; doi:10.1038/s41598-019-51576-0)
Supplement: Supplementary file 1 — Supplementary Figures and Tables [file 41598_2019_51576_MOESM1_ESM.docx]

Clonal expansion of a virulent *Streptococcus suis* serotype 9 lineage distinguishable from carriage subpopulations

### Niels Willemse^1,2^, Kees C. H. van der Ark^1,2^, Norbert Stockhofe-Zurwieden^3^, Hilde Smith^3^, Daisy I. Picavet^4^, Conny van Solt-Smits^3^, Henk J. Wisselink^3^, Constance Schultsz^1,2*^, Astrid de Greeff^3^

1. Department of Global Health-Amsterdam Institute for Global Health and Development, Amsterdam UMC, Paasheuvelweg 25, 1105 BP Amsterdam, The Netherlands
2. Department of Medical Microbiology, Amsterdam UMC, University of Amsterdam, Meibergdreef 9, 1105 AZ Amsterdam, The Netherland
3. Wageningen Bioveterinary Research, Wageningen University & Research, Houtribweg 39, 8221 RA Lelystad, The Netherlands
4. EMCA Amsterdam, Department of Medical Biology, Amsterdam UMC, University of Amsterdam, Meibergdreef 9, 1105 AZ Amsterdam, The Netherlands

*corresponding author: c.schultsz@aighd.org

# Supplementary Figures and Tables


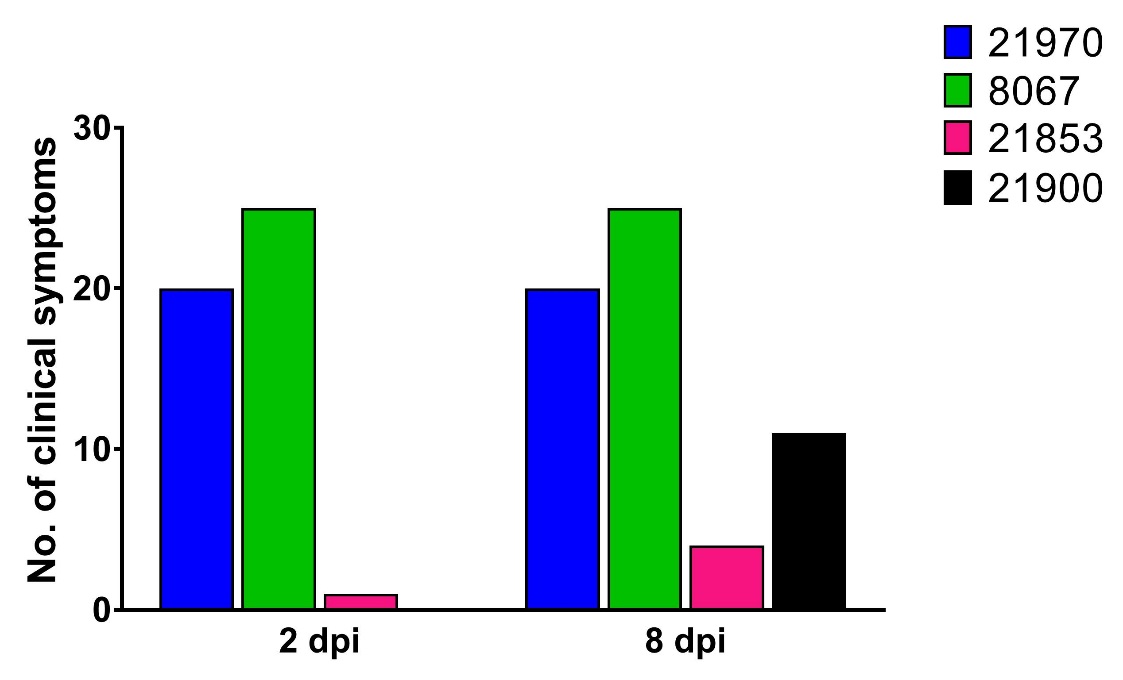


**Supplementary Figure 1A:** Count of clinical observations in virulence study after 2 days (left) and 8 days (right) in the animal experiment. Number of clinical observations did not change for the clinical isolates after 2 days, because all animals for these groups died or were euthanized after two days.


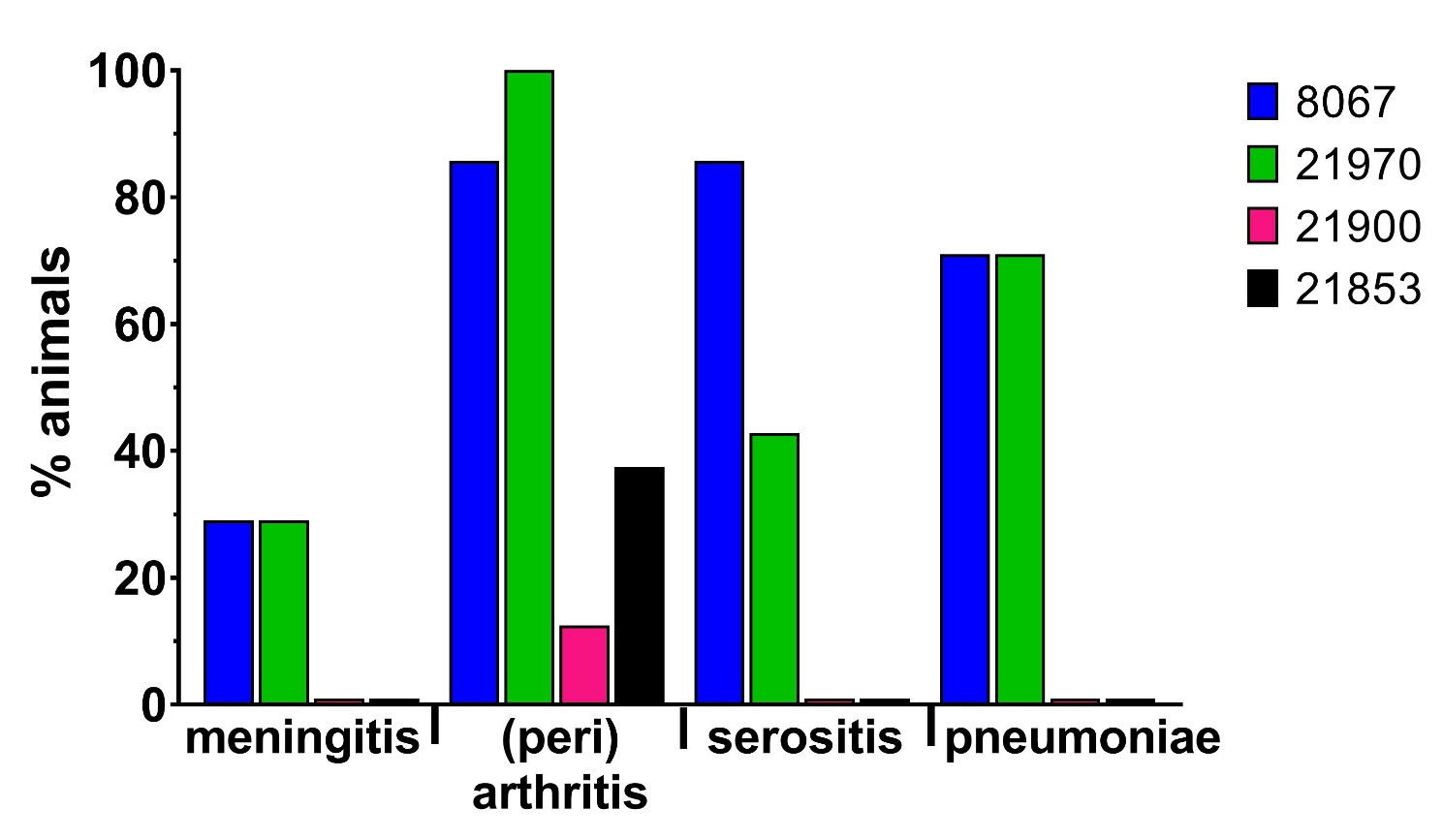


**Supplementary** **Figure 1B:** Pathological and histopathological analysis revealed inflammatory changes in the meninges of the CNS, in joints (peri-arthritis), of the serosae of the peritoneal, pleural cavities and in the lungs (pneumoniae); these changes were more often observed in pigs inoculated with the clinical isolates 8067 and 21970 (n = 7)than in piglets inoculated with the carrier isolates 21900 and 21853 (n = 8). Each bar represent the proportion of animal of a group of pigs inoculated with one of the experimental *S. suis* isolates.


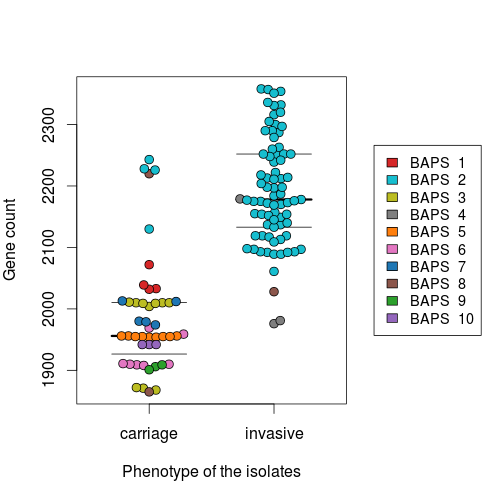


**Supplementary Figure 2:** Gene content differences between isolates with a carriage and invasive phenotype. Each isolate is represented by a dot and the color indicates the BAPS population group to which it belongs. Four BAPS 2 isolates were labelled carriage, but appear to belong to the invasive isolates gene content wise as well. The thick lines indicate the means and the thinner lines indicate the first and third quartiles. There was a significant difference between the groups using a Mann-Whitney U test (p<<0.001).

**
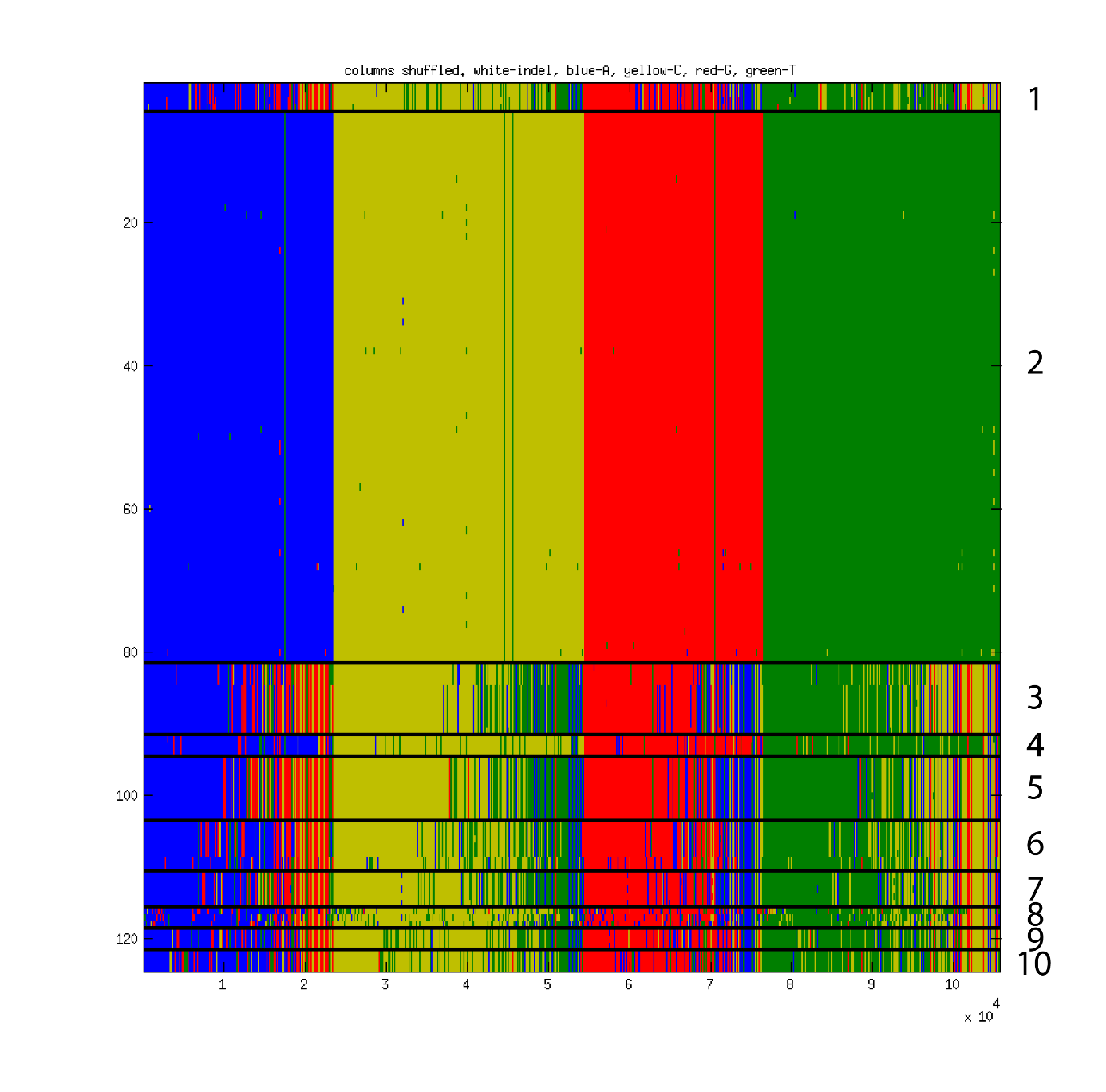
**

**Supplementary Figure 3:** Shuffled nucleotide alignment of SNPs in the core genome of 124 serotype 9 isolates. The colors are representative of each nucleotide and are indicated in the legend. Each line represents an isolate and BAPS clustered them in 10 groups which were numbered from top to bottom. BAPS group 2 contains the majority (77/124) of the isolates which belong to ST16 or are single locus variants of ST16. BAPS group 8 consists of 3 isolates which are clustered together, but are not similar in their core genome.


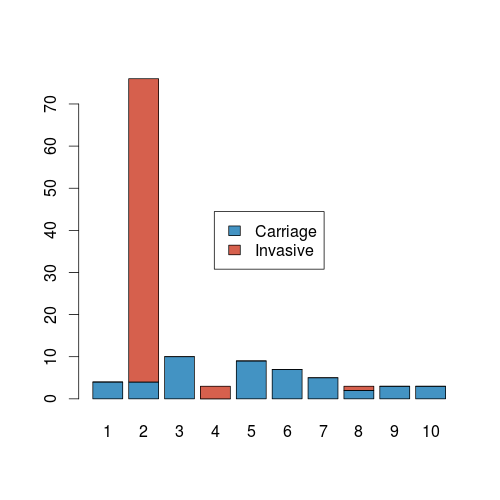


**Supplementary Figure 4:** Distribution of isolates with carriage and invasive phenotypes over the BAPS population groups. BAPS group 2 and 4 are considered the invasive groups, whilst BAPS groups 1, 3, 5-7, 9 and 10 consist solely of carriage groups. BAPS group 8 was considered a ‘rest’-group and consisted of both carriage and invasive groups.


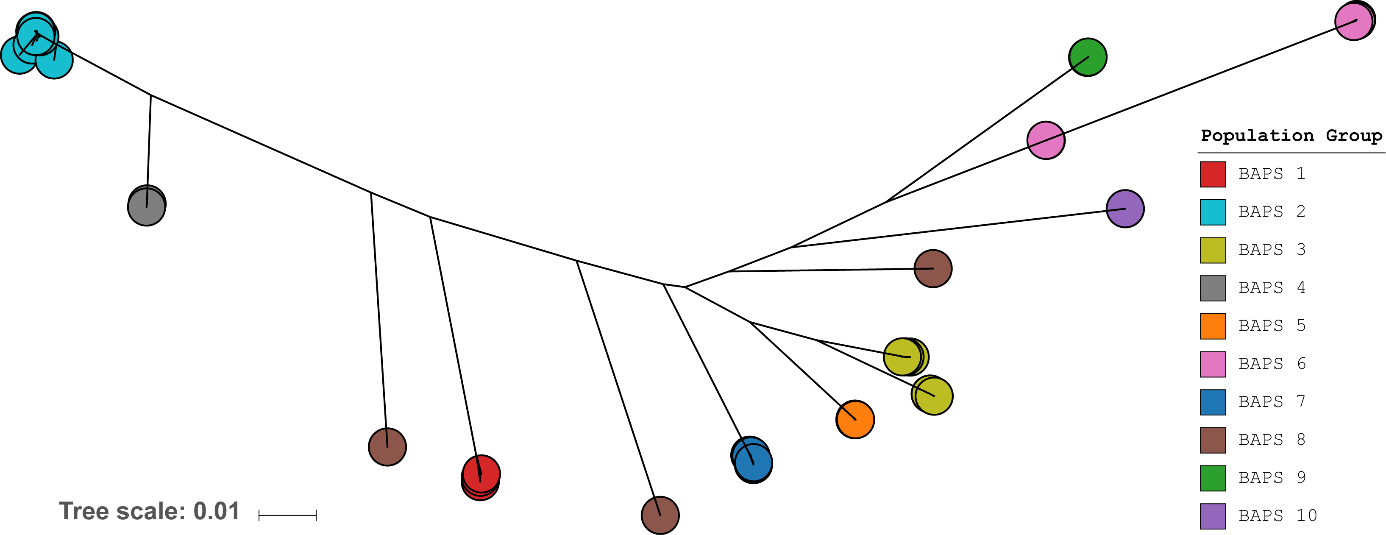


**Supplementary Figure 5:** An unrooted representation of the phylogenetic tree of Figure 4. Farm origin and phenotype metadata is lost here, but it is more illustrative of the diversity among the carriage BAPS groups.

**
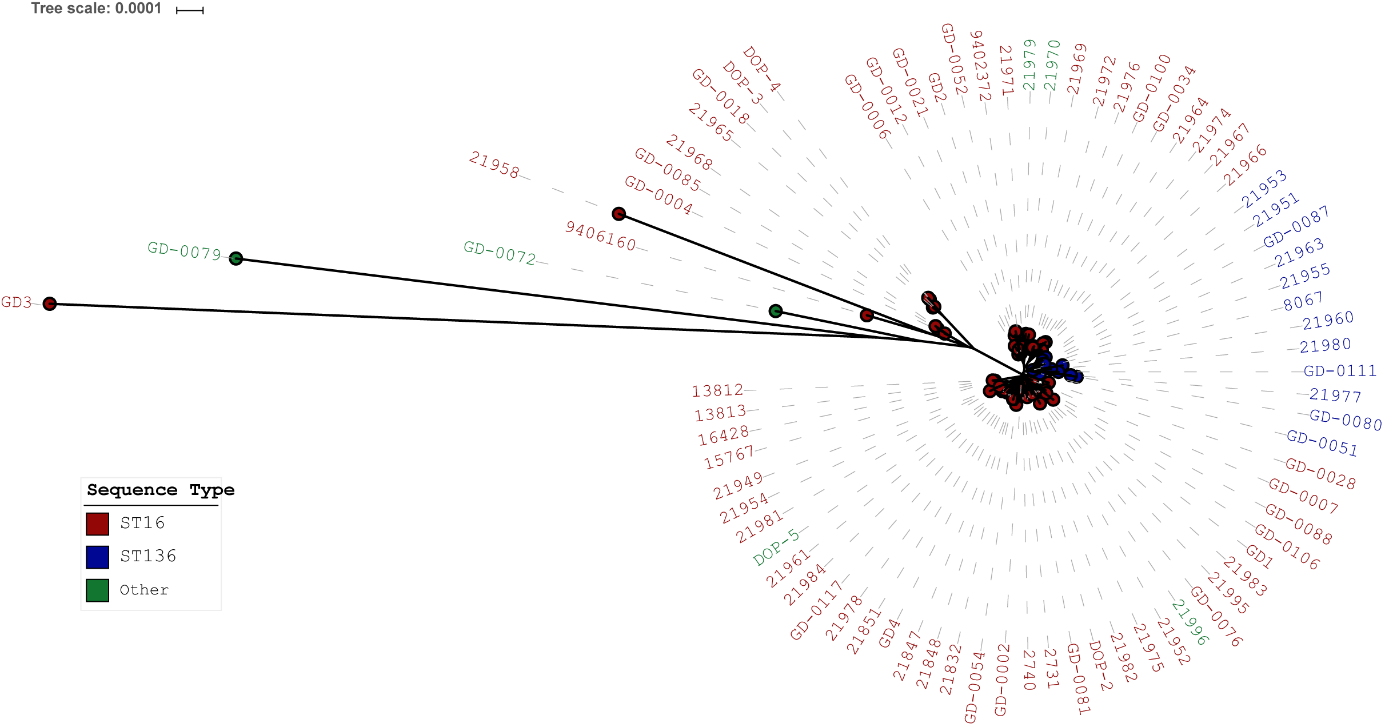
**

**Supplementary Figure 6:** Maximum likelihood tree of all isolates belonging to BAPS group 2 based on SNPs in the core genome alignment. The two major STs 16 and 136 are indicated and the minor STs are grouped in ‘other’. All STs in this tree are single locus variants of ST16. Whilst ST136 isolates form a separate branch, these isolates do cluster together with the ST16 clonal expansion. In contrast, some ST16 isolates (e.g. GD3) cluster away from the clonal cluster. Isolates 8067 and 21970 were used in the animal experiments. 8067 and 21970 cluster together with the majority of ST16 and ST136 isolates, indicating they are representative of this ST16 clonal cluster.


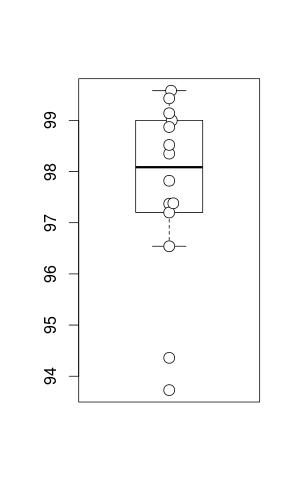


**Supplementary Figure 7:** Individual protein alignment of genes cpsA-cpsN in the capsule locus. Genes from isolates 21853 and GD2 were compared and genes cpsA (94.36%) and cpsK (93.73%) demonstrated amino acid identity of less than 95%.


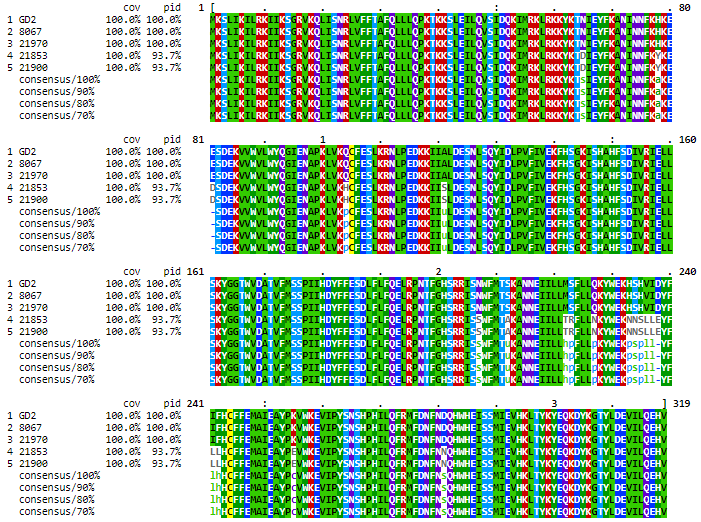


**Supplementary Figure 8:** Alignment of *cpsK* genes of invasive strains GD2, 8067 and 21970 and carriage strains 21853 and 21900. Differences can be observed between amino acid position 200 and 250. Image created with Mview (ref Brown, N.P., Leroy C., Sander C. (1998). MView: A Web compatible database search or multiple alignment viewer. Bioinformatics. 14 (4):380-381)

**
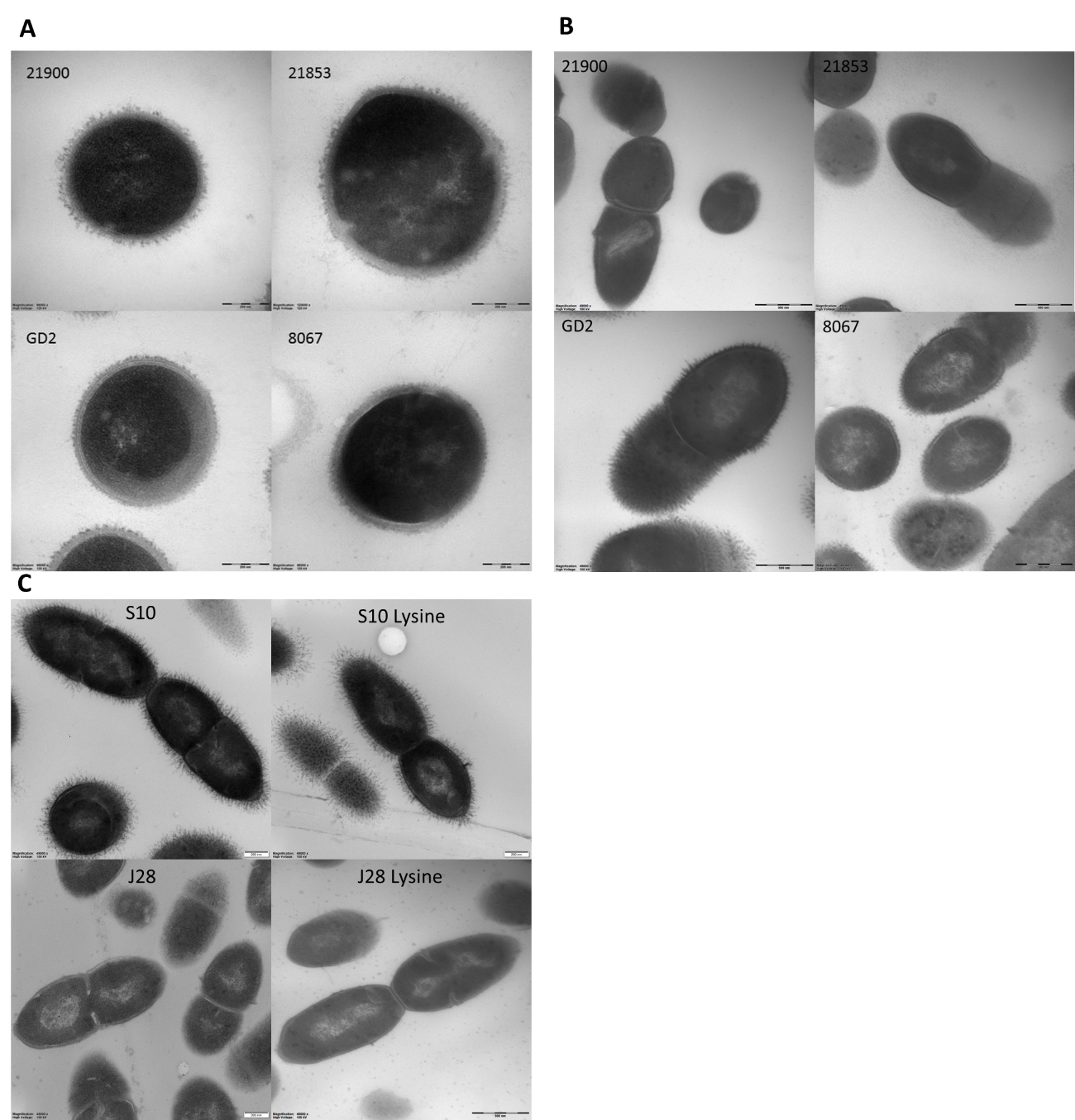
Supplementary Figure 9:** Representative electron micrographs of 2 carriage strains (top, 21853 and 21900) and 2 invasive strains (bottom, GD2 and 8067) used in *in vivo* experiments. **(A)** Isolates fixed with McDowell’s fixative, scale bar is 200 nm. All isolates show a capsule layer. **(B)** Isolates fixed with McDowell’s fixative and lysine acetate, scale bar is 500nm. Only virulent isolates show capsule layers. **(C)** Control isolates *S. suis* S10 and J28 with and without the addition of lysine acetate. S10 shows a capsule with both fixatives, the non-encapsulated J28 with neither fixative.

**Supplementary Table 1**

List of isolates used for whole genome analysis.

| **Isolate** | **Status pig** | **Isolated from** | **Farm^*^** | **Year** |
| --- | --- | --- | --- | --- |
| 21895 | Carrier | Tonsil | 4 | 2010 |
| 21899 | Carrier | Tonsil | 4 | 2010 |
| 21900 | Carrier | Tonsil | 4 | 2010 |
| 21895b | Carrier | Tonsil | 4 | 2010 |
| 21832 | Carrier | Tonsil | 2 | 2010 |
| 21847 | Carrier | Tonsil | 2 | 2010 |
| 21848 | Carrier | Tonsil | 3 | 2010 |
| 21851 | Carrier | Tonsil | 3 | 2010 |
| 2731 | Diseased | CNS | 8 | 2016 |
| 2740 | Diseased | CNS | 8 | 2016 |
| 13812 | Diseased | Liver | 9 | 2014 |
| 13813 | Diseased | Liver | 9 | 2014 |
| 15767 | Diseased | Liver | 9 | 2014 |
| 16428 | Diseased | Liver | 9 | 2014 |
| 21949 | Diseased | CNS | GD | 2011 |
| 21952 | Diseased | CNS | GD | 2011 |
| 21954 | Diseased | CNS | GD | 2011 |
| 21958 | Diseased | CNS | GD | 2011 |
| 21961 | Diseased | CNS | GD | 2011 |
| 21964 | Diseased | CNS | GD | 2011 |
| 21965 | Diseased | CNS | GD | 2011 |
| 21966 | Diseased | CNS | GD | 2011 |
| 21967 | Diseased | CNS | GD | 2011 |
| 21968 | Diseased | CNS | GD | 2011 |
| 21969 | Diseased | CNS | GD | 2011 |
| 21971 | Diseased | CNS | GD | 2011 |
| 21972 | Diseased | CNS | GD | 2011 |
| 21974 | Diseased | CNS | GD | 2011 |
| 21975 | Diseased | CNS | GD | 2011 |
| 21976 | Diseased | CNS | GD | 2011 |
| 21978 | Diseased | CNS | GD | 2011 |
| 21981 | Diseased | CNS | GD | 2011 |
| 21982 | Diseased | CNS | GD | 2011 |
| 21983 | Diseased | CNS | GD | 2011 |
| 21984 | Diseased | CNS | GD | 2011 |
| 21995 | Diseased | CNS | GD | 2011 |
| 9402372 | Diseased | unknown | unknown | 2006 |
| 9406160 | Diseased | unknown | unknown | 2006 |
| DOP-2 | Diseased | CNS | 10 | 2013 |
| DOP-3 | Diseased | CNS | 11 | 2014 |
| DOP-4 | Diseased | CNS | 11 | 2015 |
| GD-0002 | Diseased | unknown | GD | 1996 |
| GD-0004 | Diseased | unknown | GD | 1997 |
| GD-0006 | Diseased | unknown | GD | 1998 |
| GD-0007 | Diseased | unknown | GD | 1999 |
| GD-0012 | Diseased | unknown | GD | 2007 |
| GD-0018 | Diseased | unknown | GD | 2000 |
| GD-0021 | Diseased | unknown | GD | 2001 |
| GD-0028 | Diseased | unknown | GD | 2002 |
| GD-0034 | Diseased | unknown | GD | 2002 |
| GD-0052 | Diseased | unknown | GD | 2003 |
| GD-0054 | Diseased | unknown | GD | 2004 |
| GD-0076 | Diseased | unknown | GD | 2005 |
| GD-0081 | Diseased | unknown | GD | 2005 |
| GD-0085 | Diseased | unknown | GD | 2006 |
| GD-0088 | Diseased | unknown | GD | 2006 |
| GD-0100 | Diseased | unknown | GD | 2007 |
| GD-0106 | Diseased | unknown | GD | 2007 |
| GD-0117 | Diseased | unknown | GD | 2008 |
| GD1 | Diseased | CNS | GD | 2013 |
| GD2 | Diseased | CNS | GD | 2013 |
| GD3 | Diseased | CNS | GD | 2013 |
| GD4 | Diseased | CNS | GD | 2013 |
| 21970 | Diseased | CNS | GD | 2011 |
| 21979 | Diseased | CNS | GD | 2011 |
| 21996 | Diseased | CNS | GD | 2011 |
| GD-0079 | Diseased | Unknown | GD | 2005 |
| GD-0072 | Diseased | unknown | GD | 2005 |
| DOP-5 | Diseased | CNS | 12 | 2014 |
| 8067 | Diseased | unknown | unknown | 1996 |
| 21951 | Diseased | CNS | GD | 2011 |
| 21953 | Diseased | CNS | GD | 2011 |
| 21955 | Diseased | CNS | GD | 2011 |
| 21960 | Diseased | CNS | GD | 2011 |
| 21963 | Diseased | CNS | GD | 2011 |
| 21977 | Diseased | CNS | GD | 2011 |
| 21980 | Diseased | CNS | GD | 2011 |
| GD-0051 | Diseased | unknown | GD | 2003 |
| GD-0080 | Diseased | unknown | GD | 2005 |
| GD-0087 | Diseased | unknown | GD | 2006 |
| GD-0111 | Diseased | unknown | GD | 2007 |
| 21859 | Carrier | Tonsil | 3 | 2010 |
| 21853 | Carrier | Tonsil | 3 | 2010 |
| 21857 | Carrier | Tonsil | 3 | 2010 |
| 21864 | Carrier | Tonsil | 3 | 2010 |
| 21867 | Carrier | Tonsil | 3 | 2010 |
| 21874 | Carrier | Tonsil | 3 | 2010 |
| 21880 | Carrier | Tonsil | 3 | 2010 |
| 21791 | Carrier | Tonsil | 2 | 2010 |
| 21795 | Carrier | Tonsil | 2 | 2010 |
| 21810 | Carrier | Tonsil | 2 | 2010 |
| 21957 | Diseased | CNS | GD | 2011 |
| DOP-1 | Diseased | CNS | 13 | 2013 |
| GD5 | Diseased | CNS | GD | 2013 |
| 21903 | Carrier | Tonsil | 5 | 2011 |
| 21909 | Carrier | Tonsil | 5 | 2011 |
| 21910 | Carrier | Tonsil | 5 | 2011 |
| 21914 | Carrier | Tonsil | 5 | 2011 |
| 21920 | Carrier | Tonsil | 5 | 2011 |
| 21921 | Carrier | Tonsil | 5 | 2011 |
| 21933 | Carrier | Tonsil | 5 | 2011 |
| 21935 | Carrier | Tonsil | 5 | 2011 |
| 21941 | Carrier | Tonsil | 5 | 2011 |
| 21804 | Carrier | Tonsil | 2 | 2010 |
| 21814 | Carrier | Tonsil | 2 | 2010 |
| 21826 | Carrier | Tonsil | 2 | 2010 |
| 21801 | Carrier | Tonsil | 2 | 2010 |
| 21841 | Carrier | Tonsil | 2 | 2010 |
| 21892 | Carrier | Tonsil | 4 | 2010 |
| 21888 | Carrier | Tonsil | 4 | 2010 |
| 22009 | Carrier | Tonsil | 7 | 2011 |
| 22029 | Carrier | Tonsil | 7 | 2011 |
| 22014 | Carrier | Tonsil | 7 | 2011 |
| 22044 | Carrier | Tonsil | 7 | 2011 |
| 21999 | Carrier | Tonsil | 7 | 2011 |
| 22039 | Carrier | Tonsil | 7 | 2011 |
| 9401240 | Diseased | unknown | unknown | 2006 |
| 15S03465-7 | Carrier | Tonsil | 14^#^ | 2015 |
| 22004 | Carrier | Tonsil | 7 | 2011 |
| 22047 | Carrier | Tonsil | 7 | 2011 |
| 22019 | Carrier | Tonsil | 7 | 2011 |
| 22054 | Carrier | Tonsil | 6 | 2011 |
| 22057 | Carrier | Tonsil | 6 | 2011 |
| 22059 | Carrier | Tonsil | 6 | 2011 |

^*^ GD = GD Deventer, Animal Health Service

^#^ Farm from France

**Supplementary Table 2**

Number of tonsil swabs of sows and piglets positive in the serotype 9 specific PCR and number of swabs from PCR positive animals from which *S. suis* serotype 9 isolates could be isolated.

| Farm  No. | No. of swabs positive in *S. suis* serotype 9 PCR | | No. of PCR positive samples from which *S. suis* serotype 9 was isolated | |
| --- | --- | --- | --- | --- |
|  | No. positive sows/ No. tested | No. positive piglets/ No. tested | No. positive sows/ No. tested | No. positive piglets/ No. tested |
| 1 | No samples obtained | 6/40 | No samples obtained | 1/6 |
| 2 | 9/10 | 15/23 | 4/8 | 7/8 |
| 3 | No samples obtained | 23/40 | No samples obtained | 11/15 |
| 4 | 10/10 | 5/30 | 4/10 | 2/5 |
| 5 | 3/10 | 27/30 | 1/3 | 10/12 |
| 6 | 4/10 | 2/30 | 0/4 | 2/2 |
| 7 | 10/10 | 28/30 | 4/5 | 10/10 |
| Total | 36/50 | 106/223 | 13/30 | 43/58 |

**Supplementary Table 3**

List of isolates used for whole genome analysis. Their phenotype, ST and BAPS population group is indicated. Newly requested alleles and STs are highlighted in yellow.

| Isolate | Clinical | ST | aroA | cpn60 | dpr | gki | mutS | recA | thrA | BAPS |
| --- | --- | --- | --- | --- | --- | --- | --- | --- | --- | --- |
| 21895 | no | 811 | 48 | 307 | 19 | 14 | 16 | 32 | 27 | 1 |
| 21899 | no | 811 | 48 | 307 | 19 | 14 | 16 | 32 | 27 | 1 |
| **21900** | **no** | **811** | **48** | **307** | **19** | **14** | **16** | **32** | **27** | **1** |
| 21895b | no | 811 | 48 | 307 | 19 | 14 | 16 | 32 | 27 | 1 |
| 21832 | no | 16 | 5 | 17 | 5 | 12 | 1 | 10 | 4 | 2 |
| 21847 | no | 16 | 5 | 17 | 5 | 12 | 1 | 10 | 4 | 2 |
| 21848 | no | 16 | 5 | 17 | 5 | 12 | 1 | 10 | 4 | 2 |
| 21851 | no | 16 | 5 | 17 | 5 | 12 | 1 | 10 | 4 | 2 |
| 2731 | yes | 16 | 5 | 17 | 5 | 12 | 1 | 10 | 4 | 2 |
| 2740 | yes | 16 | 5 | 17 | 5 | 12 | 1 | 10 | 4 | 2 |
| 13812 | yes | 16 | 5 | 17 | 5 | 12 | 1 | 10 | 4 | 2 |
| 13813 | yes | 16 | 5 | 17 | 5 | 12 | 1 | 10 | 4 | 2 |
| 15767 | yes | 16 | 5 | 17 | 5 | 12 | 1 | 10 | 4 | 2 |
| 16428 | yes | 16 | 5 | 17 | 5 | 12 | 1 | 10 | 4 | 2 |
| 21949 | yes | 16 | 5 | 17 | 5 | 12 | 1 | 10 | 4 | 2 |
| 21952 | yes | 16 | 5 | 17 | 5 | 12 | 1 | 10 | 4 | 2 |
| 21954 | yes | 16 | 5 | 17 | 5 | 12 | 1 | 10 | 4 | 2 |
| 21958 | yes | 16 | 5 | 17 | 5 | 12 | 1 | 10 | 4 | 2 |
| 21961 | yes | 16 | 5 | 17 | 5 | 12 | 1 | 10 | 4 | 2 |
| 21964 | yes | 16 | 5 | 17 | 5 | 12 | 1 | 10 | 4 | 2 |
| 21965 | yes | 16 | 5 | 17 | 5 | 12 | 1 | 10 | 4 | 2 |
| 21966 | yes | 16 | 5 | 17 | 5 | 12 | 1 | 10 | 4 | 2 |
| 21967 | yes | 16 | 5 | 17 | 5 | 12 | 1 | 10 | 4 | 2 |
| 21968 | yes | 16 | 5 | 17 | 5 | 12 | 1 | 10 | 4 | 2 |
| 21969 | yes | 16 | 5 | 17 | 5 | 12 | 1 | 10 | 4 | 2 |
| 21971 | yes | 16 | 5 | 17 | 5 | 12 | 1 | 10 | 4 | 2 |
| 21972 | yes | 16 | 5 | 17 | 5 | 12 | 1 | 10 | 4 | 2 |
| 21974 | yes | 16 | 5 | 17 | 5 | 12 | 1 | 10 | 4 | 2 |
| 21975 | yes | 16 | 5 | 17 | 5 | 12 | 1 | 10 | 4 | 2 |
| 21976 | yes | 16 | 5 | 17 | 5 | 12 | 1 | 10 | 4 | 2 |
| 21978 | yes | 16 | 5 | 17 | 5 | 12 | 1 | 10 | 4 | 2 |
| 21981 | yes | 16 | 5 | 17 | 5 | 12 | 1 | 10 | 4 | 2 |
| 21982 | yes | 16 | 5 | 17 | 5 | 12 | 1 | 10 | 4 | 2 |
| 21983 | yes | 16 | 5 | 17 | 5 | 12 | 1 | 10 | 4 | 2 |
| 21984 | yes | 16 | 5 | 17 | 5 | 12 | 1 | 10 | 4 | 2 |
| 21995 | yes | 16 | 5 | 17 | 5 | 12 | 1 | 10 | 4 | 2 |
| 9402372 | yes | 16 | 5 | 17 | 5 | 12 | 1 | 10 | 4 | 2 |
| 9406160 | yes | 16 | 5 | 17 | 5 | 12 | 1 | 10 | 4 | 2 |
| DOP-2 | yes | 16 | 5 | 17 | 5 | 12 | 1 | 10 | 4 | 2 |
| DOP-3 | yes | 16 | 5 | 17 | 5 | 12 | 1 | 10 | 4 | 2 |
| DOP-4 | yes | 16 | 5 | 17 | 5 | 12 | 1 | 10 | 4 | 2 |
| GD-0002 | yes | 16 | 5 | 17 | 5 | 12 | 1 | 10 | 4 | 2 |
| GD-0004 | yes | 16 | 5 | 17 | 5 | 12 | 1 | 10 | 4 | 2 |
| GD-0006 | yes | 16 | 5 | 17 | 5 | 12 | 1 | 10 | 4 | 2 |
| GD-0007 | yes | 16 | 5 | 17 | 5 | 12 | 1 | 10 | 4 | 2 |
| GD-0012 | yes | 16 | 5 | 17 | 5 | 12 | 1 | 10 | 4 | 2 |
| GD-0018 | yes | 16 | 5 | 17 | 5 | 12 | 1 | 10 | 4 | 2 |
| GD-0021 | yes | 16 | 5 | 17 | 5 | 12 | 1 | 10 | 4 | 2 |
| GD-0028 | yes | 16 | 5 | 17 | 5 | 12 | 1 | 10 | 4 | 2 |
| GD-0034 | yes | 16 | 5 | 17 | 5 | 12 | 1 | 10 | 4 | 2 |
| GD-0052 | yes | 16 | 5 | 17 | 5 | 12 | 1 | 10 | 4 | 2 |
| GD-0054 | yes | 16 | 5 | 17 | 5 | 12 | 1 | 10 | 4 | 2 |
| GD-0076 | yes | 16 | 5 | 17 | 5 | 12 | 1 | 10 | 4 | 2 |
| GD-0081 | yes | 16 | 5 | 17 | 5 | 12 | 1 | 10 | 4 | 2 |
| GD-0085 | yes | 16 | 5 | 17 | 5 | 12 | 1 | 10 | 4 | 2 |
| GD-0088 | yes | 16 | 5 | 17 | 5 | 12 | 1 | 10 | 4 | 2 |
| GD-0100 | yes | 16 | 5 | 17 | 5 | 12 | 1 | 10 | 4 | 2 |
| GD-0106 | yes | 16 | 5 | 17 | 5 | 12 | 1 | 10 | 4 | 2 |
| GD-0117 | yes | 16 | 5 | 17 | 5 | 12 | 1 | 10 | 4 | 2 |
| GD1 | yes | 16 | 5 | 17 | 5 | 12 | 1 | 10 | 4 | 2 |
| **GD2** | **yes** | **16** | **5** | **17** | **5** | **12** | **1** | **10** | **4** | **2** |
| GD3 | yes | 16 | 5 | 17 | 5 | 12 | 1 | 10 | 4 | 2 |
| GD4 | yes | 16 | 5 | 17 | 5 | 12 | 1 | 10 | 4 | 2 |
| **21970** | **yes** | **803** | **5** | **17** | **5** | **12** | **1** | **199** | **4** | **2** |
| 21979 | yes | 803 | 5 | 17 | 5 | 12 | 1 | 199 | 4 | 2 |
| 21996 | yes | 805 | 5 | 17 | 5 | 12 | 278 | 10 | 4 | 2 |
| GD-0079 | yes | 151 | 5 | 17 | 5 | 70 | 1 | 10 | 4 | 2 |
| GD-0072 | yes | 155 | 5 | 17 | 5 | 71 | 1 | 10 | 4 | 2 |
| DOP-5 | yes | 804 | 5 | 17 | 207 | 12 | 1 | 10 | 4 | 2 |
| **8067** | **yes** | **136** | **5** | **62** | **5** | **12** | **1** | **10** | **4** | **2** |
| 21951 | yes | 136 | 5 | 62 | 5 | 12 | 1 | 10 | 4 | 2 |
| 21953 | yes | 136 | 5 | 62 | 5 | 12 | 1 | 10 | 4 | 2 |
| 21955 | yes | 136 | 5 | 62 | 5 | 12 | 1 | 10 | 4 | 2 |
| 21960 | yes | 136 | 5 | 62 | 5 | 12 | 1 | 10 | 4 | 2 |
| 21963 | yes | 136 | 5 | 62 | 5 | 12 | 1 | 10 | 4 | 2 |
| 21977 | yes | 136 | 5 | 62 | 5 | 12 | 1 | 10 | 4 | 2 |
| 21980 | yes | 136 | 5 | 62 | 5 | 12 | 1 | 10 | 4 | 2 |
| GD-0051 | yes | 136 | 5 | 62 | 5 | 12 | 1 | 10 | 4 | 2 |
| GD-0080 | yes | 136 | 5 | 62 | 5 | 12 | 1 | 10 | 4 | 2 |
| GD-0087 | yes | 136 | 5 | 62 | 5 | 12 | 1 | 10 | 4 | 2 |
| GD-0111 | yes | 136 | 5 | 62 | 5 | 12 | 1 | 10 | 4 | 2 |
| 21859 | no | 807 | 48 | 33 | 49 | 6 | 84 | 55 | 191 | 3 |
| **21853** | **no** | **807** | **48** | **33** | **49** | **6** | **84** | **55** | **191** | **3** |
| 21857 | no | 807 | 48 | 33 | 49 | 6 | 84 | 55 | 191 | 3 |
| 21864 | no | 807 | 48 | 33 | 49 | 6 | 84 | 55 | 191 | 3 |
| 21867 | no | 807 | 48 | 33 | 49 | 6 | 84 | 55 | 191 | 3 |
| 21874 | no | 807 | 48 | 33 | 49 | 6 | 84 | 55 | 191 | 3 |
| 21880 | no | 807 | 48 | 33 | 49 | 6 | 84 | 55 | 191 | 3 |
| 21791 | no | 802 | 48 | 33 | 49 | 228 | 98 | 55 | 57 | 3 |
| 21795 | no | 802 | 48 | 33 | 49 | 228 | 98 | 55 | 57 | 3 |
| 21810 | no | 802 | 48 | 33 | 49 | 228 | 98 | 55 | 57 | 3 |
| 21957 | yes | 147 | 5 | 1 | 5 | 12 | 1 | 10 | 1 | 4 |
| DOP-1 | yes | 819 | 243 | 1 | 5 | 12 | 1 | 10 | 1 | 4 |
| GD5 | yes | 819 | 243 | 1 | 5 | 12 | 1 | 10 | 1 | 4 |
| 21903 | no | 810 | 48 | 306 | 49 | 6 | 61 | 55 | 48 | 5 |
| 21909 | no | 810 | 48 | 306 | 49 | 6 | 61 | 55 | 48 | 5 |
| 21910 | no | 810 | 48 | 306 | 49 | 6 | 61 | 55 | 48 | 5 |
| 21914 | no | 810 | 48 | 306 | 49 | 6 | 61 | 55 | 48 | 5 |
| 21920 | no | 810 | 48 | 306 | 49 | 6 | 61 | 55 | 48 | 5 |
| 21921 | no | 810 | 48 | 306 | 49 | 6 | 61 | 55 | 48 | 5 |
| 21933 | no | 810 | 48 | 306 | 49 | 6 | 61 | 55 | 48 | 5 |
| 21935 | no | 810 | 48 | 306 | 49 | 6 | 61 | 55 | 48 | 5 |
| 21941 | no | 810 | 48 | 306 | 49 | 6 | 61 | 55 | 48 | 5 |
| 21804 | no | 808 | 57 | 308 | 40 | 6 | 98 | 15 | 193 | 6 |
| 21814 | no | 808 | 57 | 308 | 40 | 6 | 98 | 15 | 193 | 6 |
| 21826 | no | 808 | 57 | 308 | 40 | 6 | 98 | 15 | 193 | 6 |
| 21801 | no | 808 | 57 | 308 | 40 | 6 | 98 | 15 | 193 | 6 |
| 21841 | no | 808 | 57 | 308 | 40 | 6 | 98 | 15 | 193 | 6 |
| 21892 | no | 817 | 242 | 308 | 30 | 14 | 280 | 200 | 80 | 6 |
| 21888 | no | 817 | 242 | 308 | 30 | 14 | 280 | 200 | 80 | 6 |
| 22009 | no | 809 | 48 | 33 | 66 | 228 | 279 | 55 | 192 | 7 |
| 22029 | no | 809 | 48 | 33 | 66 | 228 | 279 | 55 | 192 | 7 |
| 22014 | no | 809 | 48 | 33 | 66 | 228 | 279 | 55 | 192 | 7 |
| 22044 | no | 809 | 48 | 33 | 66 | 228 | 279 | 55 | 192 | 7 |
| 21999 | no | 809 | 48 | 33 | 66 | 228 | 279 | 55 | 192 | 7 |
| 22039 | no | 806 | 25 | 34 | 66 | 228 | 34 | 19 | 34 | 8 |
| 9401240 | yes | 220 | 48 | 33 | 30 | 66 | 33 | 55 | 27 | 8 |
| 15S03465-7 | no | 820 | 244 | 310 | 183 | 228 | 53 | 26 | 56 | 8 |
| 22004 | no | 818 | 242 | 309 | 208 | 273 | 98 | 15 | 113 | 9 |
| 22047 | no | 818 | 242 | 309 | 208 | 273 | 98 | 15 | 113 | 9 |
| 22019 | no | 818 | 242 | 309 | 208 | 273 | 98 | 15 | 113 | 9 |
| 22054 | no | 48 | 37 | 41 | 20 | 46 | 38 | 5 | 27 | 10 |
| 22057 | no | 48 | 37 | 41 | 20 | 46 | 38 | 5 | 27 | 10 |
| 22059 | no | 48 | 37 | 41 | 20 | 46 | 38 | 5 | 27 | 10 |

**Supplementary Table 4**

Assembly statistics of sequenced isolates.

| **Strain** | **Scaffolds (#)** | **Largest scaffold (bp)** | **Genome size (bp)** | **N50 (bp)** | **GC-content (%)** | **Accession** |
| --- | --- | --- | --- | --- | --- | --- |
| 13812 | 91 | 140534 | 2177767 | 52927 | 41,16 | ERS1691681 |
| 13813 | 90 | 140534 | 2178463 | 53068 | 41,16 | ERS1691682 |
| 15767 | 92 | 140534 | 2175984 | 52980 | 41,15 | ERS1691683 |
| 15S03465-7 | 55 | 245306 | 2295210 | 123205 | 41,20 | ERS1691680 |
| 16428 | 93 | 140534 | 2173217 | 50375 | 41,15 | ERS1691684 |
| 21791 | 22 | 611316 | 2006704 | 264354 | 41,43 | ERS1691685 |
| 21795 | 23 | 611387 | 2005864 | 264354 | 41,43 | ERS1691686 |
| 21801 | 22 | 557444 | 2048058 | 230966 | 41,41 | ERS1691687 |
| 21804 | 22 | 557444 | 2048319 | 230966 | 41,41 | ERS1691688 |
| 21810 | 24 | 611389 | 2005040 | 210556 | 41,43 | ERS1691689 |
| 21814 | 22 | 558366 | 2047833 | 230721 | 41,41 | ERS1691690 |
| 21826 | 23 | 556388 | 2047167 | 230686 | 41,41 | ERS1691691 |
| 21832 | 88 | 116120 | 2266799 | 48997 | 41,15 | ERS1691692 |
| 21841 | 20 | 568681 | 2057750 | 258101 | 41,41 | ERS1691693 |
| 21847 | 86 | 115116 | 2259508 | 50376 | 41,15 | ERS1691694 |
| 21848 | 88 | 115113 | 2259565 | 50376 | 41,15 | ERS1691695 |
| 21851 | 75 | 142883 | 2184653 | 57131 | 41,30 | ERS1691696 |
| 21853 | 29 | 606689 | 2122001 | 197053 | 41,41 | ERS1691697 |
| 21857 | 25 | 665161 | 2119649 | 197431 | 41,41 | ERS1691698 |
| 21859 | 28 | 582999 | 2107519 | 176482 | 41,41 | ERS1691699 |
| 21864 | 27 | 665744 | 2121328 | 197215 | 41,41 | ERS1691700 |
| 21867 | 25 | 666140 | 2120881 | 197053 | 41,41 | ERS1691701 |
| 21874 | 25 | 665089 | 2119991 | 197431 | 41,41 | ERS1691702 |
| 21880 | 25 | 665390 | 2117608 | 194519 | 41,41 | ERS1691703 |
| 21888 | 19 | 576895 | 2107728 | 248510 | 41,50 | ERS1691704 |
| 21892 | 17 | 576935 | 2102088 | 247939 | 41,50 | ERS1691705 |
| 21895 | 37 | 626022 | 2171100 | 115970 | 41,36 | ERS1691706 |
| 21895b | 33 | 726325 | 2169954 | 131625 | 41,37 | ERS1691763 |
| 21899 | 39 | 661757 | 2187918 | 134026 | 41,24 | ERS1691707 |
| 21900 | 40 | 626250 | 2195874 | 111373 | 41,27 | ERS1691708 |
| 21903 | 19 | 359832 | 2092195 | 252430 | 41,43 | ERS1691709 |
| 21909 | 17 | 396984 | 2091005 | 262931 | 41,42 | ERS1691710 |
| 21910 | 16 | 359789 | 2091985 | 262867 | 41,43 | ERS1691711 |
| 21914 | 21 | 359663 | 2091936 | 178957 | 41,42 | ERS1691712 |
| 21920 | 18 | 308676 | 2090877 | 262931 | 41,42 | ERS1691713 |
| 21921 | 18 | 359842 | 2091803 | 262931 | 41,42 | ERS1691714 |
| 21933 | 16 | 359895 | 2091429 | 262931 | 41,43 | ERS1691715 |
| 21935 | 16 | 360106 | 2091980 | 262931 | 41,43 | ERS1691716 |
| 21941 | 17 | 308676 | 2091974 | 262931 | 41,42 | ERS1691717 |
| 21949 | 80 | 120824 | 2279834 | 56636 | 41,03 | ERS1691730 |
| 21951 | 78 | 279544 | 2330905 | 68925 | 41,11 | ERS1691731 |
| 21952 | 78 | 153226 | 2174907 | 59224 | 41,26 | ERS1691732 |
| 21953 | 110 | 241842 | 2292289 | 69782 | 40,89 | ERS1691733 |
| 21954 | 80 | 120524 | 2277430 | 53850 | 41,02 | ERS1691734 |
| 21955 | 80 | 141592 | 2236590 | 59068 | 41,10 | ERS1691735 |
| 21957 | 91 | 146411 | 2243495 | 51893 | 41,05 | ERS1691736 |
| 21958 | 72 | 226493 | 2341754 | 72610 | 41,00 | ERS1691737 |
| 21960 | 81 | 161153 | 2194802 | 64384 | 41,11 | ERS1691738 |
| 21961 | 81 | 131848 | 2200782 | 52743 | 41,22 | ERS1691739 |
| 21963 | 79 | 161164 | 2219912 | 64384 | 41,03 | ERS1691740 |
| 21964 | 74 | 208860 | 2200266 | 71994 | 41,07 | ERS1691741 |
| 21965 | 60 | 175656 | 2188221 | 74855 | 41,16 | ERS1691742 |
| 21966 | 84 | 208854 | 2224204 | 58712 | 41,03 | ERS1691743 |
| 21967 | 79 | 208515 | 2235917 | 64330 | 40,99 | ERS1691744 |
| 21968 | 80 | 165654 | 2360593 | 66278 | 41,06 | ERS1691745 |
| 21969 | 87 | 153676 | 2189146 | 49621 | 41,05 | ERS1691746 |
| 21970 | 86 | 131843 | 2184398 | 52090 | 41,06 | ERS1691747 |
| 21971 | 83 | 131205 | 2225732 | 55100 | 41,06 | ERS1691748 |
| 21972 | 70 | 171094 | 2143571 | 59162 | 41,20 | ERS1691749 |
| 21974 | 88 | 208795 | 2230010 | 59193 | 41,00 | ERS1691750 |
| 21975 | 67 | 141597 | 2344240 | 64387 | 41,12 | ERS1691751 |
| 21976 | 69 | 171092 | 2139805 | 60180 | 41,17 | ERS1691752 |
| 21977 | 103 | 77539 | 2163294 | 38145 | 41,16 | ERS1691753 |
| 21978 | 81 | 116565 | 2290439 | 61406 | 41,23 | ERS1691754 |
| 21979 | 82 | 131843 | 2237639 | 53389 | 41,02 | ERS1691755 |
| 21980 | 92 | 138366 | 2240209 | 52274 | 41,09 | ERS1691756 |
| 21981 | 81 | 136003 | 2187143 | 58168 | 41,05 | ERS1691757 |
| 21982 | 74 | 141617 | 2315344 | 79165 | 41,15 | ERS1691758 |
| 21983 | 65 | 242580 | 2224120 | 78927 | 41,05 | ERS1691759 |
| 21984 | 73 | 130366 | 2224285 | 64750 | 41,14 | ERS1691760 |
| 21995 | 63 | 196378 | 2303825 | 81556 | 41,13 | ERS1691761 |
| 21996 | 60 | 242677 | 2228645 | 75190 | 41,14 | ERS1691762 |
| 21999 | 28 | 365078 | 2127328 | 189628 | 41,23 | ERS1691718 |
| 22004 | 23 | 628630 | 2068210 | 241918 | 41,46 | ERS1691719 |
| 22009 | 29 | 384309 | 2156061 | 265687 | 41,15 | ERS1691720 |
| 22014 | 23 | 516339 | 2122683 | 189628 | 41,24 | ERS1691721 |
| 22019 | 20 | 628662 | 2062845 | 241859 | 41,44 | ERS1691722 |
| 22029 | 27 | 383990 | 2155503 | 267983 | 41,15 | ERS1691723 |
| 22039 | 28 | 377987 | 2043706 | 155726 | 41,43 | ERS1691724 |
| 22044 | 28 | 382481 | 2126783 | 189628 | 41,24 | ERS1691725 |
| 22047 | 23 | 628630 | 2068804 | 241744 | 41,46 | ERS1691726 |
| 22054 | 33 | 575461 | 2064991 | 160398 | 41,51 | ERS1691727 |
| 22057 | 31 | 575461 | 2064495 | 160218 | 41,51 | ERS1691728 |
| 22059 | 33 | 574555 | 2064541 | 160451 | 41,52 | ERS1691729 |
| 2731 | 67 | 242219 | 2159790 | 69328 | 41,28 | ERS1691678 |
| 2740 | 67 | 241870 | 2159972 | 69331 | 41,28 | ERS1691679 |
| DOP-1 | 84 | 130868 | 2085730 | 50320 | 41,13 | ERS1691673 |
| DOP-2 | 72 | 141596 | 2120982 | 57206 | 41,15 | ERS1691674 |
| DOP-3 | 56 | 218162 | 2217190 | 74856 | 41,19 | ERS1691675 |
| DOP-4 | 58 | 218291 | 2218895 | 74856 | 41,19 | ERS1691676 |
| DOP-5 | 102 | 130294 | 2279895 | 56543 | 41,18 | ERS1691677 |
| GD1 | 70 | 144089 | 2247524 | 64376 | 41,21 | ERS1691668 |
| GD2 | 74 | 141597 | 2254789 | 55142 | 41,16 | ERS1691669 |
| GD3 | 84 | 160878 | 2313522 | 74858 | 41,10 | ERS1691670 |
| GD4 | 84 | 161405 | 2259387 | 55525 | 41,22 | ERS1691671 |
| GD5 | 82 | 131158 | 2087015 | 51141 | 41,13 | ERS1691672 |
